# Peptidoglycan degradation machinery in *Clostridium difficile* forespore engulfment

**DOI:** 10.1111/mmi.14091

**Published:** 2018-10-30

**Authors:** Marcin Dembek, Abigail Kelly, Anna Barwinska‐Sendra, Emma Tarrant, Will A. Stanley, Daniela Vollmer, Jacob Biboy, Joe Gray, Waldemar Vollmer, Paula S. Salgado

**Affiliations:** ^1^ Institute for Cell and Molecular Biosciences, Faculty of Medical Sciences Newcastle University Newcastle upon Tyne UK; ^2^ Centre for Bacterial Cell Biology, Institute for Cell and Molecular Biosciences, Faculty of Medical Sciences Newcastle University Newcastle upon Tyne UK

## Abstract

*Clostridium difficile *remains the leading cause of antibiotic‐associated diarrhoea in hospitals worldwide, linked to significant morbidity and mortality. As a strict anaerobe, it produces dormant cell forms – spores – which allow it to survive in the aerobic environment. Importantly, spores are the transmission agent of *C. difficile* infections. A key aspect of sporulation is the engulfment of the future spore by the mother cell and several proteins have been proposed to be involved. Here, we investigated the role of the SpoIID, SpoIIM and SpoIIP (DMP) machinery and its interplay with the SpoIIQ:SpoIIIAH (Q:AH) complex in *C. difficile*. We show that, surprisingly, SpoIIM, the proposed machinery anchor, is not required for efficient engulfment and sporulation. We demonstrate the requirement of DP for engulfment due to their sequential peptidoglycan degradation activity, both *in vitro* and *in vivo*. Finally, new interactions within DMP and between DMP and Q:AH suggest that both systems form a single engulfment machinery to keep the mother cell and forespore membranes together throughout engulfment. This work sheds new light upon the engulfment process and on how different sporeformers might use the same components in different ways to drive spore formation.

## Introduction

*Clostridium difficile, *a spore forming Gram‐positive strict anaerobe, is a major cause of human morbidity and mortality in hospitals and the main cause of hospital‐acquired diarrhoea. *C. difficile* infections (CDI) place considerable economic pressure on healthcare systems, costing an estimated €3B per annum in the EU (Aguado *et al*., 2015) and $4.8B in the USA (Lessa *et al*., 2015). CDI results from gut dysbiosis, typically caused by antibiotic therapy disrupting the normal microbiota. However, current therapy for acute disease involves the use of one of three antibiotics: vancomycin, metronidazole or fidaxomycin (Silva *et al*., 1981; Teasley *et al*., 1983; Louie *et al*., 2011) that further promote dysbiosis, leaving patients acutely sensitive to reinfection or disease relapse. The recent emergence of more virulent strains with greater antibiotic resistance, more serious disease symptoms, progression and higher relapse rates (Hunt and Ballard, 2013), highlights the urgent need to develop more targeted therapeutic approaches that have minimal impact on the normal gut microbiota. One of the main issues in combating CDI and developing targeted therapeutics is the still limited understanding of *C. difficile *pathogenicity, particularly at the molecular level. Clinical symptoms are largely attributed to the much‐studied toxins; however, other aspects of the unique *C. difficile* pathobiology remain understudied.

Spores are the key infectious agent in CDI. Asporogenous *C. difficile* mutants cause disease but fail to sustain persistent infections and transmission (Deakin *et al*., 2012). Spores released from infected patients or animal hosts remain in the environment due to their resistance to common disinfectants, high temperatures and radiation (Setlow, 2007). Therefore, transmission by spores is a crucial determinant of CDI. Importantly, spores are also involved in disease persistence (Deakin *et al*., 2012), leading to recurring episodes, which affect 15–35% of patients, and present a particular risk for the elderly (Asempa and Nicolau, 2017). Despite recent studies (Fimlaid *et al*., 2013; Pereira *et al*., 2013; Saujet *et al*., 2013; Saujet *et al*., 2014; Fimlaid *et al*., 2015; Serrano *et al*., 2016) and the importance of sporulation to CDI, spore formation mechanisms in *C. difficile* remain unclear, with most of our understanding derived from work carried out in the model Gram‐positive sporeformer *Bacillus subtilis*.

**Figure 1:**
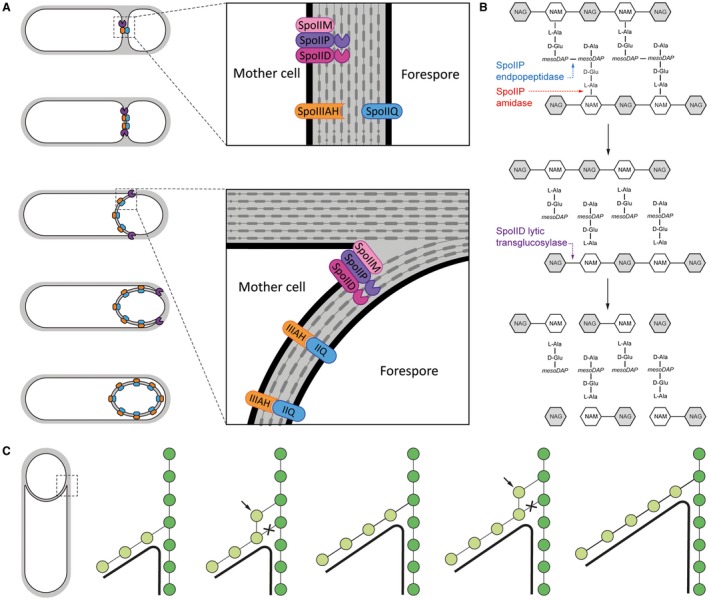
Molecular basis of engulfment. (A) Schematic representation of engulfment, as proposed for *B. subtilis*. (i) Following asymmetric cell division, components of the SpoIIDMP engulfment machinery (purple) are recruited to the septal midpoint, anchoring themselves within the mother cell membrane. (ii) The complex is then involved in thinning septal peptidoglycan as the machinery relocates to the leading edge of the engulfing membrane, while SpoIIQ (blue) and SpoIIIAH (orange) co‐localize at the mother cell/forespore interface. (iii) During engulfment, the SpoIIDMP complex ‘pulls’ the mother cell membrane around the forespore while being trailed by SpoIIQ and SpoIIIAH which interact within the intermembrane space, preventing membrane retraction. (iv) After completion of engulfment, the SpoIIDMP complex dissociates while the SpoIIQ and SpoIIIAH form a scaffold for assembly of a secretion system that spans the intermembrane space and ‘nurtures’ the spore. Adapted from Sogaard‐Andeersen, 2013. (B) Sequential breakdown of PG by SpoIID and SpoIIP. (i) SpoIIP endopeptidase activity removes peptide crosslinks while its amidase activity removes peptide stems from the glycan chain. (ii) The denuded glycans are then processed by SpoIID’s lytic transglycosylase activity, releasing NAG‐anhydroNAM disaccharides. NAG: N‐Acetylglucosamine; NAM – N‐acetylmuronic acid. (C) Cell cross‐section with glycan strands in the plane perpendicular to the long axis of the cell. One strand from old cell wall (dark green) and one strand from newly synthesized spore‐cell wall (light green) are used as a template for new glycan insertion. Coordination between glycan insertion (arrow) and peptide cross‐link degradation (cross) drives the engulfing membrane (black) forward. Adapted from Ojkic *et al*., 2016.

Spore formation is a complex differentiation programme, initiated by the master regulator Spo0A. The process begins with asymmetric cell division (Stragier and Losick, 1996; Piggot and Hilbert, 2004). The mother cell membrane then begins to engulf the forespore, transforming it into a free protoplast surrounded by two membranes of opposing polarity and isolated from the environment (Fig. 1A), nurtured by the mother cell. Once the forespore is fully engulfed, the spore coat layers are assembled, completing spore morphogenesis. Finally, the mother cell lyses and the mature spore is released (McKenney *et al*., 2013). In *B. subtilis*, forespore maturation is governed and regulated by an activation cascade of four cell‐type specific RNA polymerase *σ*‐factors (*σ* ^F^ *→* *σ* ^E^ *→* *σ* ^G^ *→* *σ* ^K^) coordinated between the forespore (*σ* ^F^, *σ* ^G^) and the mother cell (*σ* ^E^, *σ* ^K^). Cell‐cell communication allows coordination of gene expression programmes and nurturing of the forespore, which is isolated from the environment (Losick and Stragier, 1992; Stragier and Losick, 1996; Kroos *et al*., 1999; Rudner and Losick, 2001).

Several studies have focused on the driving forces of engulfment in *B. subtilis*, with two key components identified: the peptidoglycan (PG) degradation machinery formed by the mother cell proteins SpoIID, SpoIIM and SpoIIP (hereby referred to as DMP for simplicity), and the so‐called ‘zipper’ complex formed by the mother cell SpoIIIAH and the forespore SpoIIQ proteins (referred to as Q:AH), (Fig. 1A). After asymmetric cell division, SpoIIM is recruited first to the septal midpoint, which triggers localization of SpoIIP and finally SpoIID, assembling the DMP machinery anchored to the mother cell membrane (Chastanet and Losick, 2007). Peptidoglycan thinning by the DMP machinery at the leading edge of the advancing membrane has been proposed to be the driving force for engulfment, pulling the mother cell membrane around the forespore (Abanes‐De Mello *et al*., 2002). Q:AH is proposed to act as a ‘zipper’ to maintain the integrity of the double‐membrane system and help drive forward movement of the engulfing membranes (Broder and Pogliano, 2006), while other studies indicate that it can form a channel that allows communication between the mother cell and the forespore (Meisner *et al*., 2008). However, the exact function of this complex remains unclear and it could play distinct roles in different organisms (Morlot and Rodrigues, 2018). Recently, Ojkic *et al*. (2016) have proposed a detailed engulfment mechanism, where forespore‐mediated new PG synthesis, coupled with PG degradation by the mother cell DMP machinery, allows the leading edge membranes to move forward through entropic forces rather than pulling or pushing motions (Ojkic *et al*., 2016) (Fig. 1C).

In *B. subtilis*, mutation of any of the DMP proteins results in disruption of septal thinning and membrane migration, leading to characteristic membrane bulges toward the mother cell (Abanes‐De Mello *et al*., 2002). SpoIIP has been shown to possess amidase activity, cleaving the peptide stem from the *N‐*acetylmuramic acid (Mur*N*Ac) residue, and DD endopeptidase activity that hydrolyses the peptide crosslinks within PG (Morlot *et al*., 2010). SpoIID is a lytic transglycosylase that cleaves the links between Mur*N*Ac and *N*‐acetylglucosamine (Glc*N*Ac) on ‘denuded’ glycan strands (lacking the peptide stem), releasing short glycan moieties that terminate with a 1,6‐anhydro‐Mur*N*Ac residue (Morlot *et al*., 2010). SpoIIM, a predicted integral membrane protein, is not known to have an enzymatic activity but acts as an anchor for SpoIID and SpoIIP, bringing them together to act sequentially: SpoIIP first hydrolyses the peptide crosslinks and cleaves peptides from the glycan strands, releasing the denuded strands, which are the substrate for SpoIID (Fig. 1B). Recently, the structure of SpoIID has been determined, revealing the key catalytic residue and a potential ligand recognition mechanism required for activity *in vitro*, possibly involving a zinc binding site (Nocadello *et al*., 2016).

The first detailed studies of sporulation in *C. difficile* revealed important deviations from the *B. subtilis* model, particularly in the programs of gene expression in the forespore and mother cell. In *C. difficile*, gene expression relies on alternative *σ*‐factor activation pathways, resulting in less tightly coupled controls between the forespore and mother cell (Fimlaid *et al*., 2013; Saujet *et al*., 2013; Pereira *et al*., 2013), and involves different genes (Fimlaid *et al*., 2013; Saujet *et al*., 2013). Despite the conservation of the key components, it is clear that their regulation and activity can differ between sporeformers. A number of recent studies in *C. difficile* have demonstrated that both SpoIIQ and SpoIIIAH are essential for engulfment, as absence of either protein results in cells arrested at an early to intermediate stage of engulfment (Dembek *et al*., 2015; Fimlaid *et al*., 2015; Serrano *et al*., 2016). Strikingly, in our previous work we showed that strains carrying mutation of either *spoIIQ* or *spoIIIAH* exhibited significant morphological defects in the septal region, with inverted septa and membrane bulging (Serrano *et al*., 2016), a phenotype characteristic of the loss of the DMP PG degradation machinery in *B. subtilis* (Abanes‐De Mello *et al*., 2002; Morlot *et al*., 2010).

Here, we extend our previous work on the engulfment mechanism in *C. difficile* by investigating the role of the DMP orthologues (Fig. S1), their interactions with the Q:AH complex and how their enzymatic activities relate to their role in sporulation. We show that *spoIID* and *spoIIP*, but not *spoIIM*, are essential for engulfment. Indeed, strains carrying deletions of *spoIID* or *spoIIP* are arrested at early stages of engulfment, with no membrane collapse or bulging observed. Surprisingly, absence of either protein does not affect localization of either SpoIIQ or SpoIIIAH. Nevertheless, SpoIID and SpoIIQ seem to interact, as tested by bacterial two‐hybrid system, suggesting a possible localization dependence or cooperation between the two machineries. Finally, we show that catalytic point mutants of both SpoIID and SpoIIP inhibit sporulation which correlates with a lack of PG degradation enzymatic activity *in vitro*. Taken together, our results suggest a unique organization with specific roles of the DMP machinery and the Q:AH ‘zipper’ in *C. difficile *engulfment.

## Results

### *630*△erm *spo*IID and *spo*IIP mutants are defective in sporulation

Previous work using a forward genetic screen of gene essentiality in *C. difficile* indicated the importance of the DMP machinery in sporulation (Fig. S2) (Dembek *et al*., 2015), but this has not been confirmed using reverse genetics or studied further in any detail. Therefore, we set out to investigate the role of *spoIIDMP* by creating null mutants in all three components of the putative DMP complex in *C. difficile* 630△*erm*, an erythromycin‐sensitive derivative of the sequenced clinical isolate 630 (Hussain *et al*., 2005) using Allele‐Coupled Exchange (ACE) (Heap *et al*., 2012; Ng *et al*., 2013) (see details in Experimental Procedures and Fig. S3). Correct integration of mutated alleles was confirmed by PCR using primers flanking the *spoIID, spoIIM* and *spoIIP *loci (Fig. S3B) and 630△*erm*△*pyrE*‐derived mutants were restored to uracil prototrophy before further analysis. For *spoIID* and *spoIIM* mutants complementation was carried out under native promoters, whilst *spoIIP* was placed under an inducible *P_tet_* promoter (Fagan and Fairweather, 2011) to bypass presumed toxicity in *E. coli* that prevented successful cloning using the native promoter. In this case, gene expression in *C. difficile* was driven, when necessary, by addition of the inducing agent anhydrotetracycline (ATc).

**Figure 2:**
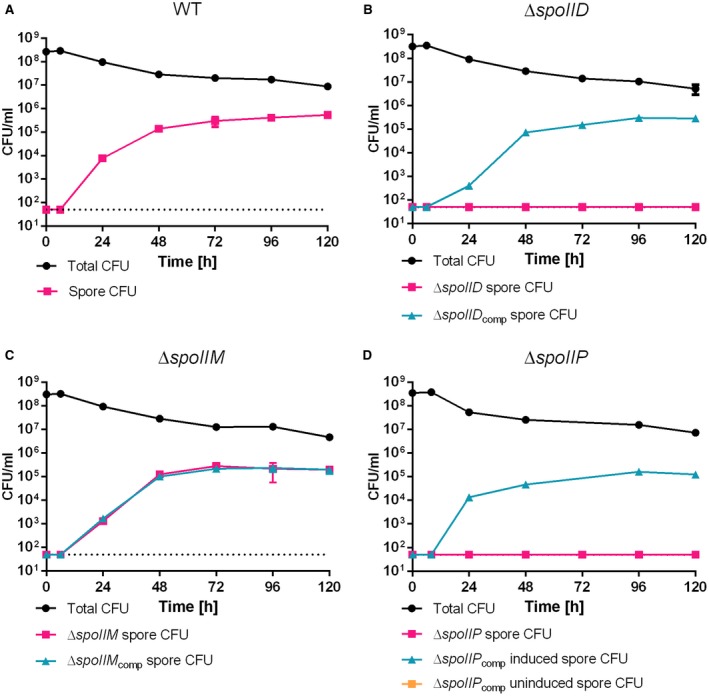
Sporulation efficiency of 630△*erm* *spoIIDMP *mutants in nutrient medium shows that *spoIID *and *spoIIP*, but not *spoIIM* are required for sporulation. Spores were undetectable for *△spoIID* (B) and *△spoIIP* (D), while normal sporulation was observed for WT (A) and *△spoIIM* (C). Complementation resulted in restored spore formation to WT levels (cyan lines). BHIS cultures were grown to OD_600_ ~0.6, sub‐cultured 1:10,000 in fresh medium and allowed to reach stationary phase (t0). Samples were removed at the indicated time points, serially diluted in sterile, pre‐reduced PBS and plated onto BHIS agar supplemented with 0.1% Taurocholate (Tch) with (spore CFU) or without (total CFU) prior heat‐treatment at 70°C for 30 min. Colonies were enumerated after 24 h of incubation under anaerobic conditions. Data presented as means ± SD from experiments performed in biological triplicate. Dotted line denotes the limit of detection of the assay (5 × 10^1^ CFU).

To test whether deletions in 630△*erm* *spoIIDMP* have an effect on sporulation, all three mutants, along with an isogenic wild type (WT) strain, were grown in BHIS broth and the number of total and heat‐resistant (spore) colony forming units (CFU) was determined over a period of 120 h. This is typically regarded as the time after which the sporulation process of a *C. difficile *culture is complete (Burns and Minton, 2011). In line with previous results, WT spores were not detected during the first 24 h, after which the number increased gradually, reaching maximum (5.3 × 10^5^ ± 2.9 × 10^4^ CFU/ml) at 120 h (Fig. 2A, Table S1). In contrast, while the deletion of *spoIID* or *spoIIP* did not affect vegetative growth (Fig. S4), both mutants were unable to produce any spores (Fig. 2B and D, TableS1). Importantly, in both cases, spore titres were restored to WT levels upon complementation (Fig. 2D, Table S1). Interestingly, and in contrast to what has been reported for *B. subtilis* (Smith *et al*., 1993), the *spoIIM* mutant formed normal amounts of spores (Fig. 2C, Table S1), as has been suggested by high throughput mutagenesis data (Fig. S2) (Dembek *et al*., 2015), highlighting the differences between sporulation mechanisms in *C. difficile* and *B. subtilis*.

### Strains carrying *spoIID* and *spoIIP* mutations are arrested at an early stage of engulfment

**Figure 3:**
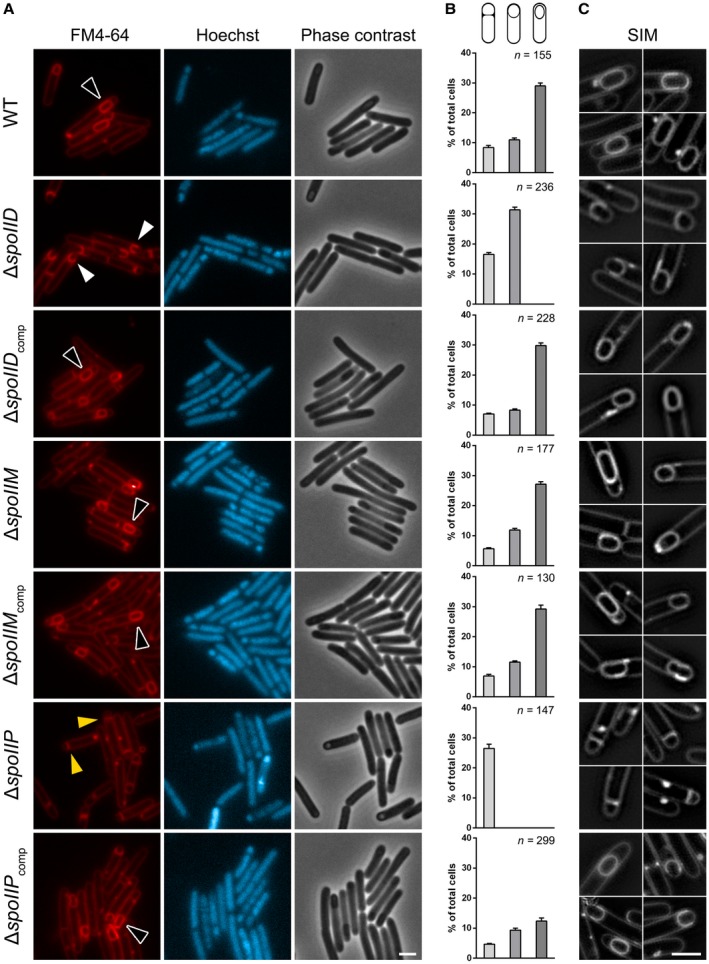
△*spoIID *and △*spoIIP* are arrested in early engulfment as seen in microscopical analysis of sporulation defects in 630△*erm* *spoIIDMP* mutants. (A) The *spoIID *mutant is seen arrested at early to intermediate stage of engulfment (white arrows) while the *spoIIP* mutant is arrested at the asymmetric division stage (yellow arrows). Fully engulfed forespores (black arrows) as seen in WT were restored in both mutants upon complementation. No significant sporulation defects could be observed for the *spoIIM* mutant. Phase contrast and widefield fluorescence microscopy images of WT and mutant cells harvested after 14 h of growth in SM broth, stained with FM4‐64 (membrane) and Hoechst 33258 (DNA). Scale bar corresponds to 2 μm. (B) Quantitative analysis of sporulating cells (stage 1: cells with flat septa; stage 2: cells with curved septa; stage 3: cells with fully engulfed forespores and mature spores). Data presented as mean ± SD from 10 random fields of view. Differences observed at each stage between the *spoIID *and *spoIIP* mutants and wild type were statistically highly significant (one‐way ANOVA, *p* < 0.00001). No significant differences were observed between the wild type and the *spoIIM *and complemented strains, apart from complementation of the *spoIIP* mutant, were a field of view with high cell density lead to observed differences, particularly at stage 3. (C) SIM images of wild type and mutant sporangia stained with FM4‐64. Scale bars corresponds to 2 μm.

To determine the stage at which mutations in *spoIIDMP* caused a sporulation defect, cultures of the three mutants and the parental WT strain were grown in sporulation media (SM) broth for 14 h, stained with a membrane dye (FM4‐64) and a DNA marker (Hoechst 33258) and imaged. At this time point, all of the main stages of sporulation, including phase‐bright spores, are represented in a *C. difficile* culture and can be quantified (Pereira *et al*., 2013). WT cells that have undergone asymmetric cell division were classified into three main groups, representing stages in the spore differentiation pathway: (i) stage 1: cells with flat septa (8.4 ± 0.7% of total cells), (ii) stage 2: cells with curved septa (11 ± 0.6%); (iii) stage 3: fully engulfed forespores and mature spores (29 ± 1%) (Fig. 3A and B). All mutants readily produced cells in stage 1 with stained forespore chromosomes indicating that neither asymmetric division or segregation and condensation of the forespore chromosomes were affected (Fig. 3A and B). However, in contrast to WT, △*spoIID* and △*spoIIP* cells failed to produce fully engulfed forespores or mature spores. In the *spoIID* mutant, most cells were arrested at an early to intermediate stage of the engulfment sequence showing flat (16.5 ± 0.6%) or partially curved (31.4 ± 0.9%) septa (Fig. 3A and B). The sporulation defect observed in the *spoIIP* mutant was even more striking. All cells that had undergone asymmetric division were arrested at an early stage of engulfment, showing flat septa (100%) (Fig. 3A and B). Importantly, all sporulation defects were alleviated upon complementation (Fig. 3A and B). In line with the results in Fig. 2C, the *spoIIM* mutant did not suffer any sporulation defects and the morphology and sporulation efficiency was indistinguishable from that of the WT (Fig. 3A and B).

Mutations in *spoIIDMP* in *B. subtilis* (Meyer *et al*., 2010) and in *spoIIQ* or *spoIIIAH* in *C. difficile* (Serrano *et al*., 2016) have been associated with septal bulging as disruption of PG hydrolytic activity, necessary for remodelling the septum during engulfment, causes newly synthesized PG to protrude into the mother cell compartment. To better visualize the asymmetric septum and the forespore membranes during engulfment and confirm whether this was also true for *spoIIDMP* mutant sporangia, cells were analyzed using Structured Illumination Microscopy (SIM) following FM4‐64 staining. Surprisingly, the characteristic septal bulging was not observed and no significant differences in the morphology of the septum were observed between WT and the arrested mutant cells (Fig. 3C). This observation raises the possibility that, in *C. difficile,* there is a yet unknown compensatory mechanism involving Q:AH or other systems that prevents bulging in the absence of SpoIIDP PG degradation activity.

### Disruption of *spoIIDMP* does not affect localization of the SpoIIQ:SpoIIIAH complex during early engulfment

**Figure 4:**
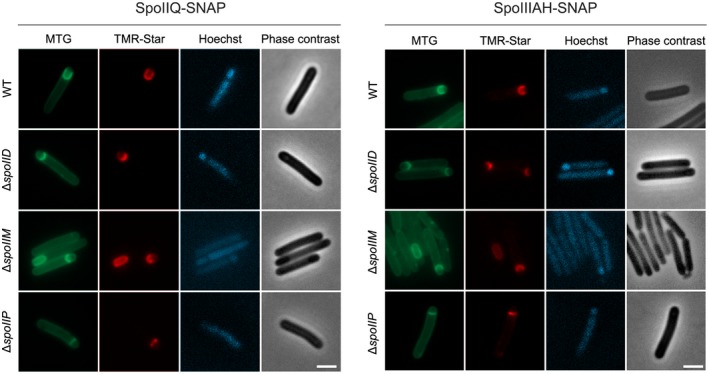
Subcellular localization of SpoIIQ and SpoIIIAH is unaffected in *spoIIDMP* mutants at early stages of engulfment. SpoIIQ and SpoIIIAH are recruited to the forespore:mother cell interface in the *spoIIDMP* mutants, along the flat septa or lining the curvature of the engulfment membrane, as described in wild type cells. However, as *spoIID *and *spoIIP *are arrested at early stages of engulfment, it is possible that DP could have a role in guiding their localization later in the process. Phase contrast and widefield fluorescence microscopy of WT and mutant cells expressing either SpoIIQ (left) or SpoIIIAH (right) SNAP fusions, harvested after 14 h of growth in SM broth and stained with MitoTracker Green (membrane), Hoechst 33258 (DNA) and TMR‐Star (SNAP substrate). Scale bars corresponds to 2 μm. Images are representative of at least three biological replicates.

Previous work in *B. subtilis* has indicated that SpoIID and SpoIIP may facilitate septal thinning to promote the localization of SpoIIQ to the septal side of the forespore membrane for its stable interaction with SpoIIIAH (Fredlund *et al*., 2013; Rodrigues *et al*., 2013). To assess whether disruption of *spoIIDMP *affects localization of SpoIIQ and SpoIIIAH, both proteins were expressed with an N‐terminal SNAP reporter (Pereira *et al*., 2013; Serrano *et al*., 2016), expressed *in trans *in *spoIIDMP* mutants and imaged by fluorescent microscopy after staining with a membrane dye (MitoTracker Green), a DNA marker (Hoechst 33258) and a fluorescent SNAP substrate (TMR‐Star). We previously showed that SpoIIQ and SpoIIIAH localize along the flat septa at the asymmetric cell division site, and later follow the curvature of the engulfing membrane around the entire contour of the forespore during engulfment (Serrano *et al*., 2016). In addition, SpoIIQ was enriched at the leading edges of the engulfing membranes, forming foci that disappear upon completion of engulfment (Serrano *et al*., 2016). The correct localization at the forespore‐mother cell interface was maintained in our *spoIID*, *spoIIM* or *spoIIP* mutants as shown for SpoIIQ‐SNAP or SpoIIIAH‐SNAP by wide field microscopy (Fig. 4) or SIM (Fig. S5). However, as the *spoIID* and *spoIIP* mutants are arrested at early stages of engulfment, we cannot exclude a role of the DP machinery in maintaining correct Q:AH localization as the membrane migrates to engulf the forespore. Regardless, it is possible that system redundancy means delocalization at early stages is only observed when multiple components of the two machineries are disrupted, as seems to be the case in *B. subtilis* (Fredlund *et al*., 2013; Rodrigues *et al*., 2013).

### BACTH analysis of interactions between components of DMP and Q:AH complexes

**Figure 5:**
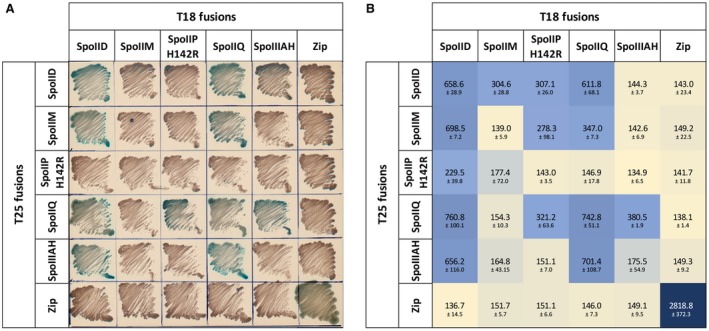
BACTH analysis of protein‐protein interactions reveals interplay between components of the DMP and Q:AH complexes. BACTH revealed that SpoIID interacts with all other proteins and itself, whilst SpoIIP only interacted weakly with SpoIID, SpoIIQ and SpoIIM. Importantly, both proteins were found to interact with SpoIIQ and partially with SpoIIIAH, indicating an interplay between both systems. (A) Observed colony growth on solid plates for the different combinations of DMP and Q:AH proteins, with blue colonies indicating interaction between the different components. *E. co*li BTH101 were transformed with pUT18C and pKT25‐based plasmids encoding full length components of DMP and Q:AH complexes fused to the T18 and T25 fragments of adenylate cyclase (CyaA). Transformed cells were plated onto selective medium and incubated for 24 h to screen for complementation of the *cya* phenotype indicating a positive interaction (blue colonies). (B) Cultures of transformed BTH101 expressing the indicated fusion proteins were screened for β‐galactosidase activity. Plasmids encoding the GCN4 leucine zipper fused to T18 and T25 fragments of CyaA were used together as positive control and in combination with other plasmids as negative control. Results are presented as means ± SD from experiments carried out in biological duplicate.

Given that we were unable to identify any clear co‐dependency in localization between the components of the DMP and Q:AH complexes by microscopy, we set out to probe protein‐protein interactions within these complexes using the Bacterial Adenylate Cyclase Two‐Hybrid (BACTH) system that allows identification of interactions within the context of the cell membrane (Karimova *et al*., 1998). To this end, full length components of both complexes were fused to the C‐terminus of either the T18 or T25 fragment of adenylate cyclase (*cyaA*) catalytic domain, expressed in a △*cya* background and probed in tandem for complementation (see Experimental Procedures for details) (Fig. 5A). The interactions were then quantified by measuring the activity of β‐galactosidase, expressed from the *lacZ* reporter. We could not obtain fusions with the native SpoIIP protein but readily obtained fusions with a putative catalytic site mutant (H142R). This mutation is not expected to interfere with localization and/or the outcome of the assay as a single amino acid substitution does not seem to affect the overall protein stability (Fig. S6) and, therefore, any possible interactions with partner proteins.

Importantly, our BACTH analysis confirmed the previously reported interactions between SpoIIQ and SpoIIIAH (Serrano *et al*., 2016) (701.4 ± 108.7 Miller Units, MU), and we observed the proposed SpoIIQ self‐interaction (Levdikov *et al*., 2012; Meisner *et al*., 2012) (742.8 ± 51.1 MU) (Fig. 5B), validating the use of BACTH to study the interactions between DMP and Q:AH. Out of all proteins tested, SpoIID had the highest number of interactions, showing strong self‐interaction (658.6 ± 28.9 MU), strong interactions with SpoIIM (698.6 ± 7.2 MU) and SpoIIQ (760.8 ± 100.1 MU), and a weaker interaction with SpoIIP_H142R_ (229.4 ± 39.8 MU). These interactions were detected irrespective of whether SpoIID was bound to the T18 or the T25 fragment of CyaA, adding to the robustness of the results. In addition, a strong interaction was detected between SpoIID and SpoIIIAH (656.3 ± 116 MU) but only when they were fused to T18 and T25 fragments respectively, despite similar amounts of protein being detected regardless of fragment fusion (Fig. S7A and D). However, SpoIIIAH fusions seem to be partially unstable, as the unfused protein is also detected (Fig. S7A and D), which could affect β‐galactosidade activity measurements. Detection of the interactions in only one direction suggest that, in this case, the orientation of the proteins within the membrane context is likely to influence protein–protein interactions. Weak interactions were also detected for SpoIIP_H142R_:SpoIIM; SpoIIP_H142R_:SpoIIQ and SpoIIQ:SpoIIM. No interactions were detected when components of the DMP and Q:AH complexes were paired with an unrelated protein (GCN4 leucine zipper) (Fig. 5A and B). Importantly, not all proteins seem to be expressed at similar levels and, in some cases, orientation appears to influence protein stability (Fig. S7). SpoIIP_H142R_ and SpoIIIAH fusions with either T18 or T25 appear to be partially unstable as both fused and unfused fragments are detected in immunoblots (Fig. S7). Conversely, SpoIIQ T18 and T25 fragments show different electrophoretic migration patterns, despite both exhibiting some level of degradation (Fig. S7). SpoIID fusions seem to be the most stable, which could explain the fact that interactions between this protein and the other components were more readily detected. Despite these caveats, as all fusion proteins are expressed in all combinations tested, we can infer that interactions observed or undetected are likely to be biologically relevant and that, in some cases, protein orientation has a clear effect in the ability to interact with other members of the engulfment machinery. These results indicate a potentially different organization of the DMP machinery from the current model proposed in *B. subtilis* (Chastanet and Losick, 2007). Notably, these observations reveal a link between components of the DMP machinery and the Q:AH ‘zipper’ which might be relevant for engulfment.

### SpoIID and SpoIIP metal binding ability

The crystal structures of SpoIID from *B. anthracis* and *C. difficile* identified critical residues and the structural basis of transglycosylase activity (Nocadello *et al*., 2016). The authors proposed that a turn‐β‐turn motif, found only in *C. difficile *SpoIID, with two cysteines (C140 and C146) and two histidines (H134 and H145), would coordinate a metal ion (Fig. S1A and B) However, despite the authors’ assignment as a zinc‐binding motif, the identity of the ion was not verified experimentally. Sequence conservation between *B. subtilis* SpoIIP and the zinc‐binding amidase CwlV, that allowed the original identification of the amidase catalytic residues (Chastanet and Losick, 2007), also suggests the possibility that these residues in SpoIIP might bind zinc (Fig. S1A).

**Figure 6:**
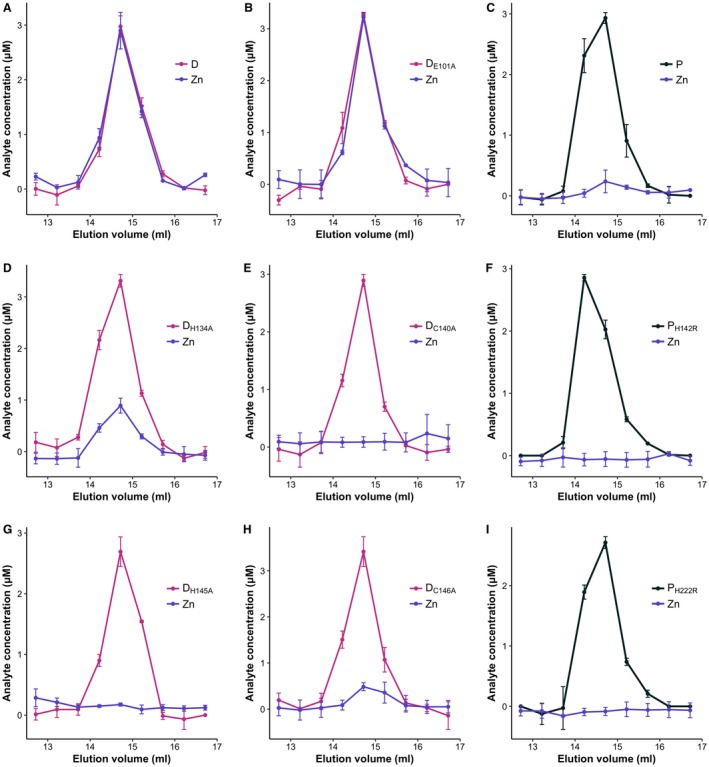
Metal content analysis of SpoIID_26‐354 _and SpoIIP_27‐339 _ Presence of zinc was confirmed in WT SpoIID_26‐354 _(A), while no ion was detected in SpoIIP_27‐339_ (C). Analysis of the SpoIID catalytic mutant E101A (B) showed no effect in the ability to coordinate zinc, whilst mutating C140 (E) or H145 (G) to alanine seems to prevent zinc binding. Surprisingly, the H134A and C146A mutants retained some zinc coordinating capability (D, H). Purified protein was analyzed by SEC with metal content (purple lines) and protein concentration (black lines) in the resulting fractions determined by ICP‐MS and absorbance at 280 nm respectively. Results are presented as mean values ± SD from two biological replicates.

In order to confirm the presence and identity of metal ions in SpoIID and SpoIIP, we analyzed the metal content of pure protein by inductively coupled plasma mass spectrometry (ICP‐MS). The soluble domain of SpoIID (residues 26‐354) and SpoIIP (residues 27‐339) were expressed in *E. coli*, purified by nickel‐affinity chromatography, followed by His‐tag removal and size exclusion chromatography (SEC) (see Experimental Procedures for details). Metal content and protein concentration in the SEC fractions were quantified by ICP‐MS and absorbance at 280 nm, respectively. SpoIID was found to bind zinc at approximately 1:1 molar ratio, confirming that it is a zinc‐binding protein (Fig. 6A). The analysis was repeated in the presence of 5 mM EDTA prior to SEC and the same zinc:protein ratio was measured, showing that low concentrations of chelating agents are unable to remove the bound metal (Fig. S8A). Surprisingly, the soluble domain of SpoIIP (aa 27‐339) does not appear to contain zinc or other bound metal ion, suggesting that SpoIIP does not require a metal co‐factor (Figs 6B, S8B).

Nocadello *et al*. (2016) previously reported that mutating residues involved in the proposed zinc binding site abrogated enzymatic activity. In order to investigate whether those mutations rendered SpoIID unable to bind zinc, we repeated the metal content analysis by ICP‐MS with recombinant SpoIID where the zinc binding residues H134, C140, H145 and C146 (Fig. S1), as well as the proposed catalytic glutamate 101, had been mutated to alanine. As expected, SpoIID_E101A_ had the same 1:1 molar ratio of zinc bound to the purified wild type protein (Fig. 6C). Conversely, SpoIID_C140A_ and SpoIID_H145A_ had no detectable zinc bound (Fig. 6D and E). Surprisingly, mutating H134 or C146 did not completely remove the ability to bind the metal, as we detected bound zinc at approximately 27 and 14% occupancy, respectively (Fig. 6F). This could imply that not all residues are required for zinc binding and that partial metal binding is sufficient for activity. In order to confirm this hypothesis, we investigated the enzymatic activity of SpoIID and SpoIIP *in vitro* on purified PG. It is worth noting that mutating the zinc‐binding residues appears to have an effect on protein folding and stability *in vitro*, with C140 and H145 mutations having a more pronounced effect, as evidenced by a decrease in melting temperature of 6°C (Fig. S6). Interestingly, SpoIID_E101A_ had an increased thermal stability, even when compared to the wild type protein (Fig. S6). It is possible that the catalytic site requires some flexibility for the reaction to occur and that removing this plasticity leads to a more stable overall structure, despite resulting in an inactive enzyme.

### SpoIID and SpoIIP activity results in PG degradation

**Figure 7:**
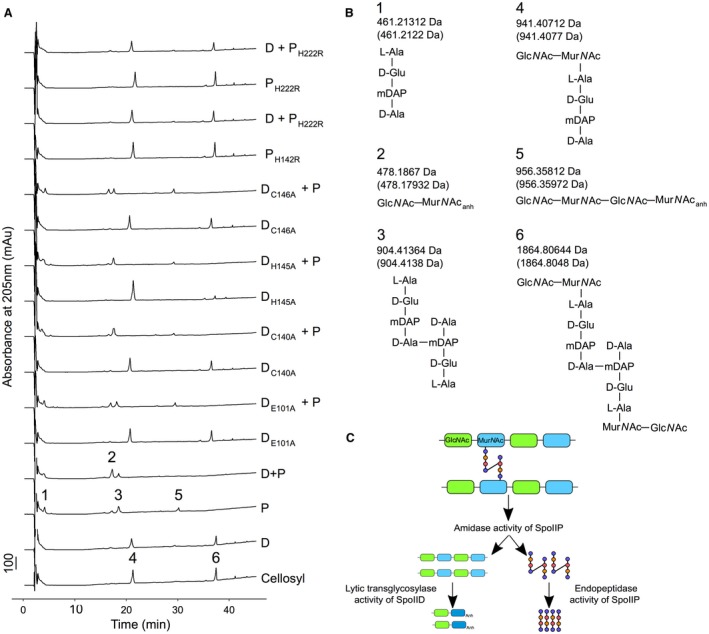
SpoIID and SpoIIP are active PG degrading enzymes. No activity is detected when SpoIID alone is incubated with purified PG, confirming the requirement of prior SpoIIP amidase activity. Only when both proteins are present do we detect the expected disaccharides and the monomer tetrapeptides. Mutating the catalytic E101 or two of the zinc binding residues, C140 or H145, results in an inactive enzyme. Conversely, substitution of H134 or C146 seems to allow the protein to retain partial activity. Finally, H142 and H222 are confirmed as key catalytic residues in SpoIIP. Enzymatic activity was assayed by analysing the products of PG digestion by the different proteins through LC‐MS, as detailed in Experimental Procedures. (A)* E. coli *BW25113△*lpp* PG was digested O/N with SpoIID, SpoIIP or their associated mutants before digestion with cellosyl. In the control reaction, PG was digested only with cellosyl. The reaction products were separated and numbered peaks of interest identified by MS. Chromatograms represent, from bottom to top: control; SpoIID; SpoIIP; SpoIID + SpoIIP; SpoIID_E101_; SpoIID_E101_ + SpoIIP; SpoIID_C140A_; SpoIID_C140A_ + SpoIIP_H145A_; SpoIID_H145A_; SpoIID_H145A_ + SpoIIP; SpoIID_C146A_; SpoIID_C146_ + SpoIIP; SpoIIP_H142R_; SpoIID + SpoIIP_H142R_; SpoIIP_H222R_ and SpoIID + SpoIIP_H222R_. (B) Proposed structures of muropeptides identified and numbered in the chromatograms in panel A. Theoretical neutral masses are given in brackets below the masses calculated from mass spectra. (C) Schematic of the sequential PG degradation activities of the SpoIIDP machinery demonstrating how the smallest products of digestion are produced.

Native or mutant isoforms of SpoIID or SpoIIP, and combinations of the enzymes, were incubated with PG purified from *E. coli*, which is rich in tetrapeptides and cross‐links formed between two tetrapeptides. This incubation was followed by digestion with the muramidase cellosyl, and analysis of the PG fragments by HPLC and mass spectrometry (see Experimental Procedures for details). SpoIID alone was unable to digest PG unless peptide‐free (denuded) strands had been created by the amidase activity of SpoIIP (Fig. 7A and B). Conversely, SpoIIP alone showed amidase activity, hydrolyzing amide bonds between the glycan chain (Mur*N*Ac) and the peptide stems, to release free tetrapeptide monomers and crosslinked tetra‐tetrapeptide dimers (Fig. 7A and B, peaks 1, 3). When incubated together, the two enzymes produced the expected products, the tetrapeptide monomers and anhydro disaccharides, as well as some tetra‐tetrapeptide dimer (Fig. 7A and B, peaks 1,2 and 3). The observed activities agree with the proposed model of sequential action of SpoIIP and SpoIID to remodel the PG at the engulfment septa (Fig. 7C). We also identified anhydro‐disaccharide and anhydro‐tetrasaccharide fragments in the presence of SpoIIP alone or with SpoIID mutants (Fig. 7A and B, peaks 2 and 5, Fig. S9A). Presumably, these are generated by the amidase activity of SpoIIP acting on the naturally occurring anhydro‐Mur*N*Ac glycan termini. These denuded anhydro strands would accumulate in our samples treated with cellosyl. Indeed, when analysing PG digested with SpoIIP but not treated with cellosyl (Fig. S9A and C), the anhydro‐saccharides are no longer visible, confirming that these sugars are the result of sequential SpoIIP‐cellosyl treatment.

The activity of SpoIID was abrogated when E101 and the zinc‐binding residues C140 and H145 were mutated to alanine, as shown by the observation that incubating PG with these mutant versions of the enzyme and SpoIIP resulted in chromatograms largely identical to those observed for SpoIIP alone (Fig. 7A). For SpoIID_C146A_, the activity was not completely abrogated as the peak corresponding to the anhydro disaccharide was more prominent than that seen in the digest with SpoIIP alone (Fig. 7A, peak 2). Mutating H134 to alanine seemingly diminishes activity in a similar way, although to a lesser extent (Fig. S9B and C).

Based on the alignment of *B. subtilis* and *C. difficile* SpoIIP protein sequences (Fig. S1), we identified H142 and H222 as putative amidase catalytic residues and tested PG degrading activity of protein variants where they were mutated to arginine (Figs 7A and B). Absence of amidase products confirmed that both residues are required for SpoIIP to cleave the peptide stems (Fig. 7A and B). In the absence of amidase activity, the products of the SpoIIP endopeptidase activity would be the disaccharide with a monomeric tetrapeptide stem (Fig. 7B, peak 4), with an accompanying reduction in the proportion of dimeric tetrapeptide present (Fig. 7B, peak 6). The fact that no differences are seen between the SpoIIP amidase point mutants and cellosyl digested PG profiles indicates that the SpoIIP endopeptidase is at least partially dependent on amidase activity.

### Disruption of SpoIID and SpoIIP enzymatic activity affects sporulation efficiency

**Figure 8:**
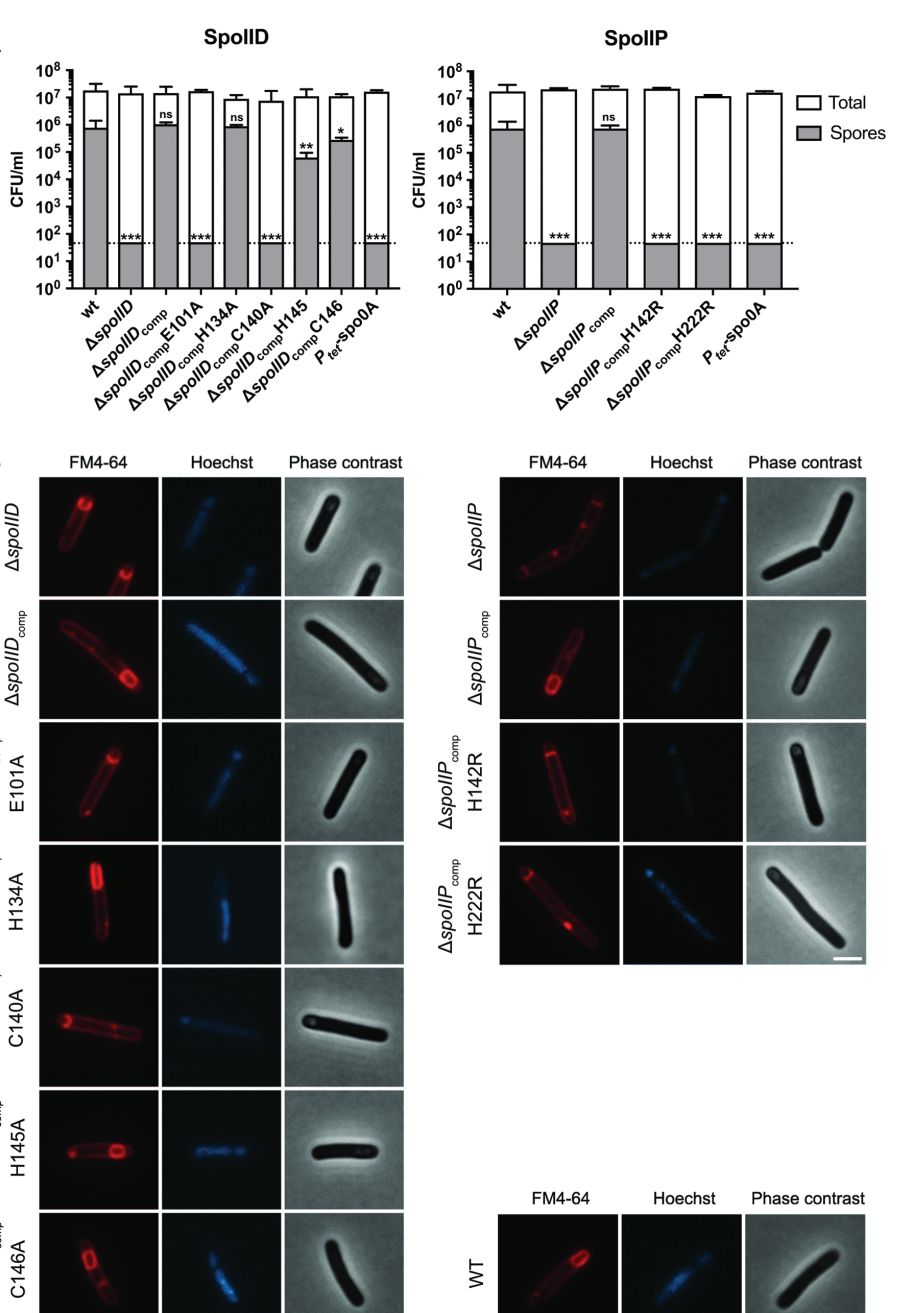
Sporulation of SpoIID and SpoIIP catalytic site mutants. (A) Endpoint sporulation efficiency assay of the different point mutants of SpoIID and SpoIIP showed that E101 and C140 in SpoIID are required for spore formation, whilst H134, H145 and C146 seem to be largely dispensable. SpoIIP H142 and H222 were confirmed to be required for sporulation, as indicated by the *in vitro* assays. BHIS cultures were grown to OD_600_ ~0.6, sub‐cultured 1:10,000 in fresh medium and allowed to reach stationary phase (t0). After 5 days, samples were removed, serially diluted in sterile, pre‐reduced PBS and plated onto BHIS agar supplemented with 0.1% Tch with (spore CFU) or without (total CFU) prior heat‐treatment at 70°C for 30 min. Colonies were enumerated after 24 h of incubation under anaerobic conditions. Data presented as means ± SD from experiments performed in biological triplicate. Dotted line denotes limit of detection of the assay (5 × 10^1^ CFU). Statistical analysis of the results was carried using one‐way ANOVA followed by Tukey’s test. ****p < 0.00001; ***p < 0.0007, **p < 0.007; *p < 0.03; ns, *p* > 0.5, for spore counts for each strain and wild type. Total CFU/ml showed no significant differences. (B) Microscopical analysis confirmed the effects of the point mutations in sporulation efficiency with E101A and C140A mutations in *spoIID*, and H142R and H222R mutations in *spoIIP* resulting in cells arrested at early engulfment. Cells carrying *spoIID* H145A or C146A mutations were indistinguishable from WT cells, forming fully engulfed spores. Phase contrast and wide field fluorescence microscopy images of cells harvested after 14 h of growth in SM broth, stained with FM4‐64 (membrane) and Hoechst 33258 (DNA). Scale bar corresponds to 2 μm.

We then sought to understand how the enzymatic activities of DP affect sporulation. To this end, site‐directed mutagenesis was used to create point mutants in the residues investigated in the *in vitro* experiments. The mutant alleles were used to complement △*spoIID* and △*spoIIP* and the sporulation efficiency of the resulting strains was analyzed as described above. In the case of SpoIID, the catalytic E101A and the zinc‐binding residue C140A substitutions rendered the protein incapable of complementing the △*spoIID* phenotype. Strikingly, the other proposed zinc‐coordinating residues had much more limited effects: H145A only caused a 5‐fold reduction in endpoint spore CFU (Fig. 8A), despite being inactive in the PG degradation assays (Fig. 7A). Consistent with the presence of zinc (Fig. 6H and D) and potential partial activity against purified PG (Fig. 7A, S9B), mutating C146 to alanine had a limited effect on sporulation efficiency, while H134A mutation resulted in sporulation efficiency equivalent to the wild type, indicating that these residues are at least partially dispensable for zinc binding and function. Importantly, the differences in stability observed *in vitro* appear to also be relevant *in vivo*, as the zinc‐binding mutated isoforms seem less abundant in extracts from sporulating cells (Fig. S10). The lower abundance of the protein could lead to the observed defects in sporulation ability. Conversely, substituting the catalytic glutamate with alanine results in a stable, inactive form that accumulates in the membrane (Fig. S10A).

In SpoIIP, both putative amidase catalytic residues H142 and H222 were found to be required, as single amino acid substitutions rendered the protein incapable of complementing the △*spoIIP* phenotype (Fig. 8A).

To confirm these results, sporulating cultures were analyzed by microscopy as described earlier. As expected, △*spoIID* cells complemented with either *spoIID*_E101A _or *spoIID*_C140A_ were phenotypically indistinguishable from the deletion mutant itself and were arrested at an early to intermediate stage of engulfment, showing either flat or curved septa with engulfing membranes reaching the forespore midpoint (Fig. 8B, left). In contrast, cells complemented with either *spoIID *H134A, *spoIID *H145A or *spoIID *C146A were able to complete the engulfment sequence as both fully engulfed forespores and mature, phase bright spores were visible. Finally, △*spoIIP* cells complemented with either *spoIIP *H142R or *spoIIP *H222R were arrested following asymmetric division, indicating that both residues are essential for the role of SpoIIP in sporulation (Fig. 8B, right).

Taken together, these findings show that the enzymatic activities of SpoIID and SpoIIP are essential for sporulation and that at least some of the SpoIID residues involved in coordinating zinc are also necessary for this role.

## Discussion

Sporulation is a critical survival strategy for strict anaerobes such as *C. difficile*, allowing them to persist in the aerobic environment. In CDI, spores are determinant for disease as the infectious agent responsible for transmission and recurrence. Despite extensive studies on sporulation mechanisms in the model organism *B. subtilis*, several molecular aspects of the pathway remain unclear. Moreover, recent studies in *C. difficile* have highlighted key differences in the mechanism of spore formation, despite the overall conservation of essential sporulation genes. Here, we show another example of how the two organisms utilize the same machineries in different ways. These observations not only expand our understanding of sporulation in *C. difficile* but also provide new insight into the role of key components during engulfment in sporeformers.

According to the current models, the mother cell proteins SpoIID, SpoIIM and SpoIIP are essential for sporulation as they provide part of the driving force for engulfment (Abanes‐De Mello *et al*., 2002; Morlot *et al*., 2010; Ojkic *et al*., 2016). Indeed, we show that deletion of either *spoIID* or *spoIIP* prevents sporulation in *C. difficile*, as expected. Moreover, cells seem to be arrested at very early stages of sporulation, soon after the formation of the asymmetric septa. This is particularly evident for *spoIIP* deletions, with all cells that initiated sporulation arrested at this stage. Conversely, some cells carrying the *spoIID* deletion seem to be able to partially initiate engulfment. As the proteins are proposed to act sequentially, with SpoIIP required to create the denuded glycan chains that are the substrate for SpoIID activity (Abanes‐De Mello *et al*., 2002; Morlot *et al*., 2010), it is unsurprising that lack of the first enzyme in the cascade has a more pronounced effect. Surprisingly, in *C. difficile*, SpoIIM seems dispensable for sporulation, suggesting that the DMP machinery might be less tightly organized in this bacterium. Alternatively, it is possible that *C. difficile* possesses as of yet unidentified mechanisms, absent in *B. subtilis,* for correct organization of SpoIID and SpoIIP that can fully compensate for the lack of SpoIIM. A third possibility is that SpoIID and SpoIIP can organize in the intersporangial space directly, without the need of SpoIIM or other components. Our BACTH results showing that SpoIID and SpoIIP can interact directly (Fig. 5) seem to support the latter hypothesis. Moreover, we detected only weak interactions of SpoIIP with SpoIIM, contradicting the current assembly model, where SpoIIM recruits SpoIIP which then recruits SpoIID. Instead, SpoIID seems to interact with SpoIIM and SpoIIP directly. Importantly, data from Ribis *et al*. (2018) shows that SpoIIP expression is regulated by the forespore‐specific *σ* ^F^ regulator and is expected to be present in the forespore membrane. Conversely, SpoIID is anchored to the mother cell membrane, with expression under the mother cell‐specific regulator *σ* ^E^, strengthening the idea that a SpoIID‐SpoIIP (DP) direct interaction across the intersporangial space could provide a simpler PG degradation machinery. If this is the case, the role of SpoIIM in organizing the enzymatic machinery could be at least partially redundant.

Strikingly, the characteristic membrane bulging, associated with incomplete PG degradation during engulfment, observed in *B. subtilis* (Abanes‐De Mello *et al*., 2002) and in *C. difficile* for *spoIIQ* and *spoIIIAH* mutants (Serrano *et al*., 2016), is absent in strains carrying *spoIID* or *spoIIP* deletions. Membrane bulging may indicate that the synthesis of new PG is faster than the degradation of old PG, leading to excessive PG/membrane that invaginates toward the mother cell. The lack of significant bulging in the *spoIID* and *spoIIP* mutants could indicate that (i) PG remodeling events are halted completely, with both synthesis and degradation being affected, or (ii) that other PG degradation mechanisms can partially compensate for the lack of these enzymes but fail to drive membrane engulfment. In the latest proposed engulfment model by Ojkic *et al*. (2016), entropic forces generated by the coordination of synthesis of new PG by forespore proteins, and degradation of old‐new PG bonds by DMP, drive engulfment in *B. subtilis*. In this scenario, the lack of activity of DP could halt the process, stalling engulfment at early stages and preventing sporulation. Alternatively, if another PG degradation system is present, it might be able to degrade newly synthesized PG but fail to break the new‐to‐old PG bonds that are presumed to drive engulfment. Either scenario is compatible with our observations, and further investigations combining deletions of forespore‐associated PG synthases with individual and double *spoIID/spoIIP* deletions are required to confirm the current model and identify the link between DP and PG remodelling activities driving engulfment.

One possible alternative PG degradation mechanism could be provided by Q:AH. In our previous work (Serrano *et al*., 2016), we found that deletion of either *spoIIQ* or *spoIIIAH* leads to membrane bulging, suggesting that PG degradation activity was affected, either directly or indirectly. If indeed SpoIIQ or the Q:AH ‘zipper’ has PG hydrolase activity, this could be restricted to newly synthesized PG, excluding the new‐to‐old PG links proposed to be important (Ojkic *et al*., 2016). In this context, the concerted action of the DP machinery and Q:AH might be required to drive engulfment in *C. difficile*, as both are essential for sporulation. This would suggest that co‐localization, or at least coordinated spatial organization, might be important for this process. Interestingly, when investigating the localization of either SpoIIQ‐SNAP or SpoIIIAH‐SNAP in a *spoIID* or *spoIIP* mutant background, we found no mislocalization evident at early stages of engulfment, with both proteins being correctly recruited to the septa. It is possible that redundant mechanisms involving all proteins in the machinery are responsible for correct organization of the engulfment apparatus, and that absence of only one component has no effect. Alternatively, other components might be involved in coordinating the spatial organization of DP and Q:AH at the septa and throughout engulfment. One way to investigate this possibility is to study localization of Q and AH in double △*spoIID/spoIIP* mutants, combined with *spoIIQ*, *spoIIIAH* deletions.

**Figure 9:**
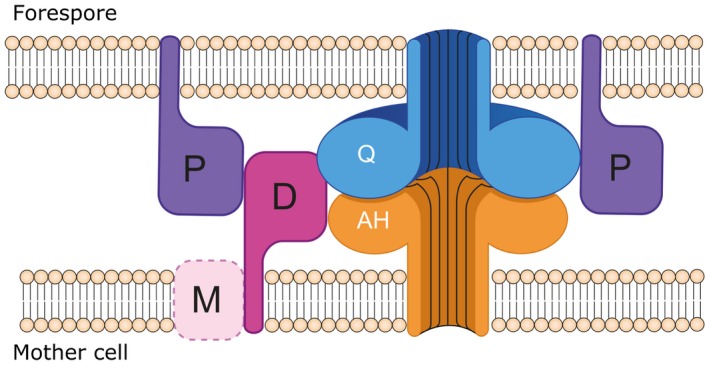
Engulfment machineries in *C. difficile. *Proposed organization of the DP and Q:AH engulfment machineries. SpoIID (D, dark pink) interacts strongly with SpoIIQ (Q, blue) and weakly with SpoIIP (P, purple) and SpoIIIAH (AH, orange). SpoIIP is anchored to the forespore membrane and establishes weak interactions with SpoIIQ and SpoIID. The role of SpoIIM (M, pink) is unclear, as it seems to be dispensable for engulfment in *C. difficile* and only interactions with SpoIID have been detected. In this new model, Q:AH could act as anchor for the DP machinery, with both systems contributing to keeping the two‐membrane system tightly together throughout engulfment. Q:AH is shown as a channel‐forming complex as proposed by Meisner *et al*. (2012) and Levdikov *et al*. (2012), although the existence of this pore has not been unambiguously demonstrated to date.

In order to further test the hypothesis that Q:AH and DMP are involved in complex, redundant connections, we investigated the potential interactions between the different proteins. As a means to preserve the native interactions as much as possible, we used a bacterial two‐hybrid system to study all pair‐wise combinations between the five proteins. The previously observed interaction between SpoIIQ and SpoIIIAH (Serrano *et al*., 2016), as well as the proposed self‐interaction of SpoIIQ (Levdikov *et al*., 2012; Meisner *et al*., 2012), were confirmed, indicating that the method is suitable for identification of interactions within these machineries. Apart from the weak interactions between SpoIID and SpoIIP, and SpoIIP with SpoIIM, we also observed significant interactions of SpoIID with SpoIIQ and SpoIIIAH which implies that the two machineries interact. This hypothesis is reinforced by the weak interactions observed between SpoIIP and Q:AH. Combining this data with the observations by Ribis *et al*. (2018), the engulfment machinery in *C. difficile *would be anchored to the forespore membrane by SpoIIP and SpoIIQ and to the mother cell membrane by SpoIID and SpoIIIAH, with SpoIIM playing an ancillary, redundant role (Fig. 9). This complex interaction network would ensure that the two‐membrane system remains together throughout engulfment and could help compensate for the lack of one of the components, ensuring that the engulfment apparatus is maintained by more than one interaction.

We then investigated the role of the enzymatic activity of SpoIID and SpoIIP *in vivo* and its effect on sporulation, as well as identifying the resulting products *in vitro*. Confirming the model proposed in *B. subtilis, *SpoIIP has amidase and endopeptidase activities, producing free monomeric peptide stems and denuded glycan chains that are the substrate for SpoIID’s lytic transglycosylase to release the disaccharide units (Fig. 7C). This coordinated activity seems to be required to drive engulfment, as mutating the putative catalytic SpoIID E101 residue or the amidase SpoIIP putative residues H142 or H222 resulted in no detectable spores and no PG degradation activity. Mutating one of the SpoIID proposed zinc binding residues (Nocadello *et al*., 2016), C140, had a similar phenotype *in vivo* and *in vitro*, which raised the possibility that metal coordination is essential for enzymatic activity and overall role in engulfment. Surprisingly, mutating H145 had limited effect in sporulation efficiency and no visible phenotype, despite the lack of activity observed in PG degradation assays and the absence of zinc. These results indicate that not all residues involved in zinc binding are important for catalysis, and/or that other mechanisms are important *in vivo*. Indeed, we have confirmed that SpoIID coordinates zinc but not all residues are essential, as SpoIID_C146A_ and SpoIID_H134A_ retained partial metal binding capability (Fig. 6) and residual activity (Fig. 7), resulting in very limited to no effect on sporulation efficiency (Fig. 8). Nocadello *et al*. (2016) suggested a role for the zinc binding motif that would allow a more rigid structure around the substrate binding groove. However, the observed interactions of H145 and C146 with the glycan chain are mediated by main chain atoms (Fig. S1B), so the mutations are less likely to disrupt these contacts. Our results indicate that mutating the zinc‐binding residues affects protein stability *in vitro *(Fig. S6) and *in vivo *(Fig. S10), suggesting an overall structural role of Zn, which could also influence substrate binding and/or recognition. Structural studies of the different mutants, in the presence or absence of the ligand and/or substrate mimics are required to further elucidate the structural and catalytic role of zinc as well as details of the enzymatic mechanism.

Our results also confirm that SpoIIP amidase activity is essential for sporulation and that H142 and H222 are the catalytic residues. Similarity with other amidases (Chastanet and Losick, 2007), where the catalytic residues are proposed to coordinate a zinc ion, raised the possibility that SpoIIP would also use the metal as a cofactor. Our work shows that the protein does not appear to bind zinc or other divalent cations with similar properties (Fig. S5B) and further studies, particularly at structural level, are required to elucidate the catalytic mechanism. Our PG degradation assays also indicate that the endopeptidase activity is at least partially dependent on the amidase activity. Ongoing work on the identification of the endopeptidase catalytic residues and analysis of its effects on sporulation will further our understanding of SpoIIP activity mechanisms and their role in engulfment.

Work presented here and by A. Ribis *et al*. (2018) shows that organization of the engulfment machinery in *C. difficile* differs from that proposed in the model organism *B. subtilis*. We propose that a DP machinery, interlinked with the Q:AH channel, provides the driving force for engulfment, with SpoIIM being largely dispensable. Moreover, some level of redundancy between the two protein systems, and possibly other yet unidentified components, leads to a more robust mechanism. Our work also provides a more detailed understanding of PG degradation at the molecular level, indicating key residues involved in catalysis. Elucidating these aspects and the key differences with currently proposed models also raises new interesting questions and hypotheses. Identification of the correct localization mechanisms of the two machineries in the absence of SpoIIM requires further investigation. One important question to address is substrate recognition by DP: if, as proposed by Ojkic *et al*. (2016), the driving force for engulfment is DP cleavage of old–new PG interactions, how does the machinery recognize these links? More detailed investigation at the molecular level will provide key insight into these and related questions, enhancing our understanding of the sporulation mechanism in Clostridia and other sporeformers.

## Experimental procedures

### Bacterial strains and growth conditions

*C. difficile* strains were grown statically at 37°C in a DG250 workstation (Don Whitley Scientific) under anaerobic conditions (10% H_2_, 10% CO_2_, 80% N_2_) in BHIS (37 g brain heart infusion, 5 g yeast extract and 1 g L‐cysteine per litre), or SM (90 g Bacto peptone, 5 g proteose peptone, 1 g NH_4_SO_4_ and 1.5 g Tris base per litre), supplemented where necessary with thiamphenicol (15 μg/ml), D‐cycloserine (250 μg/ml), fluoroorotic acid (FOA; 2 mg/ml) and uracil (5 μg/ml). *E. coli* strains were grown aerobically at 37°C with shaking in LB or TB supplemented where necessary with chloramphenicol (15 μg/ml), carbenicillin (100 μg/ml) and kanamycin (50 μg/ml). For plate cultures, media were solidified with 1.5 % (w/v) agar. A detailed list of strains used in this study is provided in Table S3.

### Molecular biology and PCR techniques

Plasmid DNA was isolated using the GeneElute Mini‐prep kit (Sigma‐Aldrich) according to the manufacturer’s instructions. *C. difficile* genomic DNA was isolated as described previously (Dembek *et al*., 2015). PCR experiments were carried out using either KOD Hot‐start DNA polymerase (Merck) or OneTaq Hot‐start DNA polymerase (NEB) according to manufacturer’s instructions. Digestion, ligation, and analysis of plasmid and genomic DNA were carried out using standard procedures. Nucleotide sequence analysis was carried out by GATC Biotech and the results were analyzed using Geneious 9.1.5 (Biomatters Ltd.). A detailed list of primers used in this study is provided in Table S2 **.**

### Construction of plasmids for Allele‐Coupled Exchange (ACE)

Plasmids used to introduce 900 bp (codons 21‐320), 450 bp (codons 21‐170) and 900 bp (codons 21‐320) in‐frame deletions in *spoIID* (CD630_0124), *spoIIM* (CD630_1221) and *spoIIP* (CD630_2469), respectively, were constructed as follows. The pMTL‐YN3 plasmid backbone was linearized by inverse PCR using primers oMLD015 and oMLD016. 1,200 bp upstream and downstream homology regions were PCR‐amplified using primers oMLD017‐oMLD018 and oMLD019‐oMLD020 (*spoIID*); oMLD021‐oMLD022 and oMLD023‐oMLD024 (*spoIIM*); oMLD025‐oMLD026 and oMLD027‐oMLD028 (*spoIIP*). The overlapping fragments were joined using the Gibson Assembly Cloning Kit (NEB) as per manufacturer’s instructions yielding pMLD090, pMLD091 and pMLD092, targeting *spoIID, spoIIM* and *spoIIP*, respectively. Plasmids used to complement the *spoIID *and *spoIIM* mutants were constructed as follows. The pMTL‐YN1C plasmid backbone was linearized by restriction digest with BamHI and NotI. *C. difficile* 630 *spoIID* and *spoIIM* alleles were PCR‐amplified using primers oMLD065‐oMLD066 and oMLD067‐oMLD068 respectively. A 150 bp 5’ non‐coding region containing the ribosome binding site (RBS) and the putative promoter signals as identified by BPROM (SoftBerry) was included for both genes. The resulting fragments were joined using the Gibson Assembly Cloning Kit (NEB) as per manufacturer’s instructions, yielding pMLD090 and pMLD091. To complement the *spoIIP* mutant, *P_tet_* regulatory elements were PCR amplified from pRPF185 (Fagan and Fairweather, 2011), using primer pair oMLD133‐oMLD134 and inserted between NotI and SacI sites in pMTL‐YN1C, yielding pMLD116. *C. difficile* 630 *spoIIP* allele containing the RBS was PCR‐amplified using primer pair oMLD244‐oMLD245 and inserted between SacI and BamHI sites in pMLD116, yielding pMLD142. A detailed list of plasmids used in this study is provided in Table S4**.**

### Construction and isolation of *spoIIDMP* mutants

All mutants and complemented strains were created in the 630△*erm*△*pyrE *background *via* allele‐coupled exchange as described previously (Heap *et al*., 2012; Ng *et al*., 2013). Plasmids carrying mutated *spoIID, spoIIM *and *spoIIP* alleles were transformed into *E. coli* CA434 and conjugated into *C. difficile *630△*erm*△*pyrE *as described previously (Purdy *et al*., 2002). Resulting colonies were restreaked twice onto fresh BHIS agar plates supplemented with 15 μg/ml thiamphenicol and 250 μg/ml d‐cycloserine to select for plasmid integration and to counter‐select for *E. coli. *Following single cross‐over, colonies were sub‐cultured onto *C. difficile* Defined Medium (CDDM) (Karasawa *et al*., 1995) agar plates supplemented with 2 mg/ml FOA and 5 μg/ml uracil before patch‐plating on BHIS agar supplemented with 15 μg/ml thiamphenicol to screen for plasmid excision. FOA‐resistant, thiamphenicol‐sensitive clones were screened by PCR to separate mutants from WT revertants using primer pairs oMLD037‐oMLD038 (*spoIID*), oMLD039‐oMLD040 (*spoIIM*) and oMLD041‐oMLD042 (*spoIIP*) (Fig. S3B).

### Complementation of *spoIIDMP* mutants

The *pyrE+* phenotype was restored by conjugating pMTL‐YN1 into the isolated mutants. The resulting colonies were sub‐cultured onto non‐supplemented CDDM agar to select for uracil prototrophy indicating successful allele exchange. Colony PCR using primers oMLD035‐oMLD036 flanking the *pyrE* locus confirmed restoration of the *pyrE* allele. In order to complement the mutants, pMLD090, pMLD091, pMLD142 and their derivatives were used instead of pMTL‐YN1 to restore the *pyrE+* phenotype and introduce a WT/mutated copy of the deleted gene immediately downstream of *pyrE* as described above.

### Sporulation assays

To ensure synchronized growth and minimize spore carry‐over, *C. difficile* cultures were grown in BHIS broth to logarithmic growth phase (OD_600_ ~0.6), diluted 1:10,000 in fresh broth, and grown overnight to stationary phase before starting the assay. Total and heat‐resistant CFUs were then enumerated at 24 h intervals for 5 days. To this end, at each time point serial dilutions were prepared in pre‐reduced PBS and 20 μl were spotted in triplicate onto BHIS agar supplemented with 0.1% (w/v) sodium taurocholate. To determine the number of spores, samples were incubated at 70°C for 30 min before serial dilution and spotting onto plates. Colonies were enumerated after 24 h incubation in an anaerobic cabinet. A similar procedure was used for final endpoint sporulation assays but samples were only collected after 120 h. Statistical analysis of the endpoint results was carried out using a one‐way ANOVA test in GraphPad Prism 7 (GraphPad Software, La Jolla, CA, USA, www.graphpad.com). Differences between total CFU/ml values are not statistically significant (*p* > 0.5), whilst significance of variations in spore CFU/ml when comparing deletion and point mutants to the wild‐type varied, with 0.003 < *p* < 0.0001 (details in Fig. 8). Complementation with wild type copy of the gene results in no significant differences both for total and spore counts (*p* > 0.05).

### Microscopy

A sample of 0.5 ml from cultures grown in SM broth was harvested by centrifugation (2 min at 4,000 × *g*) 14 h after inoculation. Cells were washed with 1 ml of PBS, resuspended in 100 μl of PBS and spotted onto 1.2% (w/v) agarose pads supplemented with a lipophilic steryl membrane dye: FM4‐64 or MitoTracker Green (Invitrogen; 1 μg/ml), a DNA dye: Hoechst 33258 (Thermo Fisher; 1 μg/ml) and 0.5% (v/v) dimethylsulfoxide (DMSO). Widefield microscopy images were captured using the Metamorph software package on a Nikon Ti microscope. SIM was performed using Nikon N‐SIM equipped with Nikon CFI APO TIRF 100/1.49 oil objective, 561 nm (Cobolt Jive 100) solid‐state lasers, and Andor Xion X3 EMCCD camera. Image capture and reconstruction of high resolution 3D SIM images was performed with NIS elements 4.0 (Nikon). The cells were immobilized on 1.2% agarose slides as described above. To reduce the binding of hydrophobic membrane dyes on the coverslip surface, which interferes with the projection of structured illumination pattern, the coverslips were plasma‐cleaned before use. All images were processed using ImageJ (Schindelin *et al*., 2012; Schneider *et al*., 2012) following capture. All microscopy is representative of at least three biological replicates.

For the analysis of sporulation stages, 10 fields of view were used to quantify cells at different stages. Statistical analysis of the results was carried out using a one‐way ANOVA test in GraphPad Prism 7. Differences between wild type and all complemented strains and for △*spoIIM* are not statistically significant (*p* > 0.5), apart from △*spoIIP*_comp_. In this case, the fact that one field of view had a much higher concentration of cells might have resulted in the variations observed, particularly in stage 3. For △*spoIID *and *△spoIIP*, differences observed in each stage are highly significant, with *p* < 0.0001.

### Bacterial Adenylate Cyclase Two‐Hybrid System (BACTH)

The Bacterial Adenylate Cyclase Two‐Hybrid System Kit (Euromedex) was used according to manufacturer’s instructions. Briefly, plasmids pUT18C and pKT25 were used to construct N‐terminal fusions with T18 and T25 fragments of adenylate cyclase for *spoIIDMP*, *spoIIQ* and *spoIIIAH*. Combinations of pUT18C and pKT25‐based plasmids were transformed into chemically competent *E. coli* BTH101 cells which were plated onto LB agar supplemented with carbenicillin (100 μg/ml), kanamycin (50 μg/ml), X‐Gal (50 μg/ml) and 0.5 mM IPTG and grown at 30°C for 24 h to allow for complementation. pUT18C‐zip and pKT25‐zip plasmids encoding the GCN4 leucine zipper fused to T18 and T25 fragments of adenylate cyclase, respectively, were used together as positive controls and in combination with other plasmids as negative controls. Experiments were carried out in duplicate.

### β‐galactosidase assay

Liquid cultures of *E. coli *BTH101 carrying various combinations of pUT18C and pKT25‐based plasmids were grown in LB supplemented with carbenicillin (100 μg/ml) and kanamycin (50 μg/ml) at 37°C O/N in a 2 ml deep well block. These were subcultured 1:50 in LB supplemented with carbenicillin (100 μg/ml), kanamycin (50 μg/ml) and 0.5 mM IPTG and grown at 30°C to OD_600_ ~0.5. Cultures were diluted 1:10 with permeabilization buffer (70 mM Na_2_HPO_4_; 30 mM NaH_2_PO_4_; 1 mM MgSO_4_; 0.2 mM MnSO_4_; 100 mM β‐mercaptoethanol; 0.002% SDS; pH 7.0) in a total volume of 1 ml and incubated for 30 min at 37°C. Reactions were developed by adding 250 μl of ONPG (4 mg/ml) and then stopped by adding 500 μl of 1M Na_2_CO_3. _β‐galactosidase activity was quantified using the following equation, where *v* equals volume of culture used in ml and *t *equals time of reaction in min. The mean value ± SD of biological duplicates is presented.

$$\beta -\text{galactosidase activity}\left[\text{Miller units}\right]=1000*\frac{Abs_{420}}{Abs_{600}*v*t}$$

### SpoIID and SpoIIP overexpression and purification

DNA fragments encoding the soluble domains of *C. difficile* 630 SpoIID (aa 26‐354) and SpoIIP (aa 27‐339) were PCR‐amplified using primer pairs oMLD212‐oMLD213 and oMLD214‐oMLD215, respectively. Resulting products were digested with NcoI and XhoI and inserted into similarly digested pETM‐11 backbone to create N‐terminal fusions with TEV‐cleavable 6xHis tags, yielding pAXK001 and pAXK002. Site‐directed mutagenesis of SpoIID and SpoIIP was carried out by inverse PCR according to standard procedures using primers listed in Table S3 to generate the SpoIID and SpoIIP point mutants reported here. Resulting plasmids were transformed into *E.coli* Rosetta (DE3) chemically competent cells (NEB) according to manufacturer’s instructions. For overexpression purposes, 1 L cultures were grown in TB supplemented with kanamycin (50 μg/ml) and chloramphenicol (30 μg/ml) at 37°C with agitation to OD 0.6–0.8. Upon reaching the desired OD, glucose was added to a final concentration of 1% (v/v), cells were induced with 1 mM IPTG and grown O/N at 18°C with agitation. Cells were harvested by centrifugation (4,000 × *g* for 20 min at 4°C), resuspended in 20 ml lysis buffer (50 mM Tris‐Cl pH 8.0, 300 mM NaCl, 20 mM imidazole, 100 μg/ml lysozyme; 10 μg/ml DNase; 1 tablet of cOmplete mini EDTA‐free protease inhibitors (Roche)), lysed by sonication (5 s pulses for a total of 10 min with cooling on ice) and centrifuged once more to separate the soluble and insoluble fractions (20,000 rpm for 30 min at 4°C in a JA 25.50 rotor). The soluble cell extract was applied onto a 5 ml HisTrap HP (GE Healthcare) pre‐equilibrated with buffer containing 50 mM Tris pH 8.0, 300 mM NaCl, 20 mM imidazole. Protein was eluted in 50 mM Tris‐Cl pH 8.0, 300 mM NaCl, 250 mM imidazole while collecting 10 ml fractions. Following SDS‐PAGE analysis, fractions containing the desired protein were pooled, supplemented with 5 mM DTT, mixed with TEV protease in a 100:1 (w/w) ratio and dialysed O/N against 5 L of 50 mM Tris‐Cl pH 8.0, 300 mM NaCl to cleave off the 6xHis tag and remove excess imidazole. The cleaved protein was reverse purified on a 5 ml HisTrap HP, concentrated on a 30,000 Da MWCO Amicon spin concentrator and applied onto a HiLoad Superdex S200 16/600 SEC column. Protein was eluted in 20 mM Tris‐Cl pH 8.0, 150 mM NaCl while collecting 2 ml fractions. Following SDS‐PAGE analysis, fractions containing the desired protein were pooled, concentrated as above and frozen at −80°C for further analysis.

### Metal content analysis

Purified protein samples in 20 mM Tris‐Cl pH 8.0, 150 mM NaCl were diluted to a concentration of 10 µM (untreated samples). Where appropriate, 5 mM EDTA was added to the samples before size exclusion chromatography. Each sample was then loaded individually onto a Superdex 200 GL 10/300 Increase column (GE Healthcare). To avoid contamination with metal and/or EDTA, all samples were analyzed in the order: EDTA followed by untreated, and the column was primed with 20 mM Tris‐Cl pH 8.0, 150 mM NaCl, 5 mM EDTA or 20 mM Tris‐Cl pH 8.0, 150 mM NaCl, as appropriate. 0.5 ml fractions were collected during elution. Samples for elemental analysis were prepared by adding 300 μl of each fraction to 2.7 ml of 2.5% HNO_3_ (Suprapur, Merck), containing 20 ppb Ag and Pt or 20 ppb In and Pt as the internal standards. Samples were loaded with an auto‐sampler (Cetac 900) onto the instrument (Thermo x‐series), operating in collision cell mode (CCT), using 3.0 ml min^−1^ flow of 8% H_2_ in He as the collision gas. Following sample ionization within an argon plasma (99.99% purity), ions with specific mass/charge ratios were quantified by comparing the number of ions colliding with the detector to a standard curve of the target ions (Mg, Mn, Cu, Co, Zn at 0, 1, 5, 10, 25, 50, 75 and 100 ppb). Samples were analyzed using the peak‐jump method (100 sweeps, 20–30 ms dwell time on 3–5 channels per isotope, separated by 0.02 atomic mass units) and compared to elemental standards. The protein concentration of each fraction was determined by absorbance at 280 nm. Two biological replicates were analyzed per sample. Measurements are presented as means ± standard deviations.

### Peptidoglycan isolation

Isolation of PG from *E. coli *BW25113△*lpp*, lacking the peptidoglycan‐bound lipoprotein Lpp (Baba *et al*., 2006), was carried out as previously described (Glauner, 1988), with slight modifications. Briefly, cells grown in LB to OD_600_ ~0.8 were harvested by centrifugation at 5,500 rpm, 4°C, resuspended in ice‐cold MilliQ and added dropwise to boiling 8% HPLC‐grade SDS, then allowed to boil for a further 30 min. SDS was removed by washing in warm MilliQ, as monitored by the Hayashi test (Hayashi, 1975). Pellets were resuspended in 10 mM Tris‐Cl 10 mM NaCl pH 7.0 with 10 mg/ml amylase (1 mg/ml final concentration) and incubated for 2 h at 37°C, at which point pronase E was added (0.10 mg/ml) and the mixture incubated for another hour. SDS solution was added to yield a final concentration of 2% SDS and samples were boiled for 15 min. SDS was removed as above and the resulting PG was stored in 0.02% sodium azide at 4°C.

### Peptidoglycan digestion and analysis by reverse‐phase high pressure liquid chromatography

Digestion reactions were prepared by incubating 10 μl PG from *E. coli *BW25113△*lpp* with the enzymes (10 μM final concentration): SpoIID, SpoIIP, SpoIID + SpoIIP, SpoIID_E101A_, SpoIID_E101A_ + SpoIIP, SpoIID_H134A_, SpoIID_H134A_ + SpoIIP, SpoIID_C140A_, SpoIID_C140A_ + SpoIIP, SpoIID_H145A_, SpoIID_H145A_ + SpoIIP, SpoIID_C146A_, SpoIID_C146A_ + SpoIIP, SpoIIP_H142R_, SpoIID + SpoIIP_H142R_, SpoIIP_H222R_, SpoIID + SpoII_H222R_. Mixtures were incubated in 10 mM Hepes pH 7.2, 50 mM NaCl, 0.05% Triton X‐100, 1 mM ZnCl_2 _for 24 h with stirring at 37°C, before digestion in the presence of 0.04 mg/ml of celloysl (kindly provided by Hoechst, Frankfurt, Germany) overnight in cellosyl buffer (80 mM sodium phosphate pH 4.8). Reactions were terminated by boiling and samples were dried (ScanVac), dissolved in 0.25 M sodium borate pH 9.0 and reduced with solid sodium borohydride. After 30 min, reduction was terminated by adjusting the pH to 3–4 with 20% HPLC‐grade phosphoric acid, before injection of 20 μl into the HPLC system (Agilent 1100, with an ACE3 C‐18AQ column (2.1 × 150 mm). RP‐HPLC conditions were as follows: buffer A: 0.1% TFA in water to a maximum of 85% buffer B (0.1% TFA in acetonitrile) over 80 min at a flow rate of 0.2 ml/min.

### Mass spectrometry analysis

Eluate from the RP‐HPLC described above was analyzed by infusion mass spectrometry (Bui *et al*., 2009) by directing it to the ion source on an LTQ‐FT mass spectrometer (Thermo). The spray voltage was set at 4.2 kV and the transfer capillary temperature at 250°C. Mass spectra were collected over the range m/z = 150–2000 with MS/MS fragmentation spectra triggered for all ion signals >5 × 10^3^ intensity.

## Author contributions

MD designed the study, carried out experiments, collected and analyzed data, wrote and revised the manuscript; AK, ABS, ET and WS carried out experiments, analyzed the data and revised the final manuscript. DV and JB helped carry out enzymatic studies and analyze the data. JG and WV designed the study, analyzed data and wrote/revised the manuscript. PSS designed the study, analyzed data, supervised the study, wrote and revised the manuscript.

## Supporting information


**Fig. S1.** Conservation of SpoIIDMP sequences between *B. subtilis* and *C. difficile*
**Fig. S2.** Forward genetic screen of *spoIIDMP* essentiality in *C. difficile* sporulation
**Fig. S3.** Construction of *spoIID*, *spoIIM* and *spoIIP* mutants in 630△*erm* by ACE
**Fig. S4.** Growth curves of *spoIIDMP* mutants
**Fig. S5.** SIM analysis of SpoIIQ‐SNAP and SpoIIIAH‐SNAP localization in *spoIIDMP* mutants
**Fig. S6.** Purity and stability of SpoIID_26‐35_ and SpoIIP_27‐339_ proteins
**Fig. S7.** Immunoblot analysis of BACTH strains
**Fig. S8.** Extended metal content analysis of SpoIID_26‐354_ and SpoIIP_27‐339_
**Fig. S9.** Peptidoglycan degradation assays
**Fig. S10.** Immunoblot analysis of *C. difficile* SpoIID, SpoIIP and point mutant strains
**Table S1.** Sporulation frequency
**Table S2.** Strains used in this study
**Table S3.** Primers used in this study
**Table S4.** Plasmids used in this study
