# Supplementary material for: Peptidoglycan degradation machinery in Clostridium difficile forespore engulfment
Source: Mol Microbiol. 2018 Oct 30;110(3):390–410. doi: 10.1111/mmi.14091 (PMC6221140; doi:10.1111/mmi.14091)
Supplement: Supplementary file 1 — Fig. S1. Conservation of SpoIIDMP sequences between B. subtilis and C. difficile Fig. S2. Forward genetic screen of spoIIDMP essentiality in C. difficile sporulation Fig. S3. Construction of spoIID, spoIIM and spoIIP mutants in 630△erm by ACE Fig. S4. Growth curves of spoIIDMP mutants Fig. S5. SIM analysis of SpoIIQ‐SNAP and SpoIIIAH‐SNAP localization in spoIIDMP mutants Fig. S6. Purity and stability of SpoIID26‐35 and SpoIIP27‐339 proteins Fig. S7. Immunoblot analysis of BACTH strains Fig. S8. Extended metal content analysis of SpoIID26‐354 and SpoIIP27‐339 Fig. S9. Peptidoglycan degradation assays Fig. S10. Immunoblot analysis of C. difficile SpoIID, SpoIIP and point mutant strains Table S1. Sporulation frequency Table S2. Strains used in this study Table S3. Primers used in this study Table S4. Plasmids used in this study [file MMI-110-390-s001.pdf]

SUPPLEMENTARY INFORMATION

Peptidoglycan degradation machinery in *Clostridium difficile* forespore engulfment

Marcin Dembek[1], Abigail Kelly[1*], Anna Barwinska-Sendra[1*], Emma Tarrant[1], Will A. Stanley[1], Daniela Vollmer[2], Jacob Biboy[2], Joe Gray[1], Waldemar Vollmer[1,2], Paula S. Salgado[1,2#]

[1]Institute for Cell and Molecular Biosciences, Faculty of Medical Sciences, Newcastle University, Newcastle upon Tyne, UK

[2]Centre for Bacterial Cell Biology, Institute for Cell and Molecular Biosciences, Faculty of Medical Sciences, Newcastle University, Newcastle upon Tyne, UK

*These authors contributed equally

[#] **Corresponding author**. Address: Institute for Cell and Molecular Biosciences, Faculty of Medical Sciences, Newcastle University, Newcastle upon Tyne, UK. Telephone: +44 (0)191 208 7432; paula.salgado@newcastle.ac.uk

Running title: Engulfment machinery in *C. difficile*

Keywords: *C. difficile*, sporulation, engulfment, DMP, peptidoglycan remodelling

Supporting results and discussion

Protein stability

Protein expression in BACTH strains

Supplementary experimental procedures

Circular dichroism

Affinity purification of polyclonal antibodies from rabbit serum

Detection of proteins using immunoblotting

List of figures and tables

Figure S1. Conservation of SpoIIDMP sequences between *B. subtilis* and *C. difficile*

Figure S2. Forward genetic screen of *spoIIDMP* essentiality in *C. difficile* sporulation

Figure S3. Construction of *spoIID, spoIIM* and *spoIIP* mutants in 630Δ*erm* by ACE

Figure S4. Growth curves of *spoIIDMP* mutants

Figure S5. SIM analysis of SpoIIQ-SNAP and SpoIIIAH-SNAP localisation in *spoIIDMP* mutants

Figure S6. Purity and stability of SpoIID$_{26-35}$ and SpoIIP$_{27-339}$ proteins

Figure S7. Immunoblot analysis of BACTH strains

Figure S8. Extended metal content analysis of SpoIID$_{26-354}$ and SpoIIP$_{27-339}$

Figure S9. Peptidoglycan degradation assays

Figure S10. Immunoblot analysis of *C. difficile* SpoIID, SpoIIP and point mutant strains

Table S1. Sporulation frequency

Table S2. Strains used in this study

Table S3. Primers used in this study

Table S4. Plasmids used in this study

Supporting results and discussion

Protein stability

Wild type and point mutant versions of SpoIID$_{25-354}$ (sSpoIID) and SpoIIP$_{27-339}$ (sSpoIIP) recombinantly expressed were obtained at a high degree of purity, as shown in immunoblotting (Figure S6A) and SDS-PAGE gels (Figure S6B). Moreover, all proteins seem stable, as no degradation was detected (Figure S6A, B). Analysis of CD spectra from 190 to 240 nm (Figure S6C) indicates all proteins are folded. However, point mutations of the zinc-binding residues of SpoIID – H134, C140, H145 and C146 – lead to reduced protein stability as reflected by reduced melting temperatures (T_m), with C140A and H145A proteins exhibiting the lowest values. This effect appears to be linked to some reduction in helical content, as calculated by CDSSTR (Figure S6C). Conversely, mutating the catalytic aspartate 101 to alanine seems to lead to increased protein stability, with T_m increasing by 3 °C. A similar stabilizing effect seems to occur when mutating catalytic residues in SpoIIP, where both mutants have a higher T_m than the wild type protein. The mutations seem to lead to decreased helical and strand content, with accompanying increase of the coiled/turn content.

Overall, it seems that, although all proteins are folded in solution, the zinc-binding mutations in SpoIID affect their relative stability which could impact both *in vitro* catalysis and *in vivo* activity. Indeed, when analyzing *in vitro* PG degradation activity, C140A and H145A proteins appear mostly inactive (Figure 7), whilst H134A and C146A retain some activity.

The varying stability could also explain the observed differences in sporulation efficiency (Figure 8) so we investigated the presence of functional SpoIID (A, C) and SpoIIP (B, D) in strains of *C. difficile* where isogenic mutants were complemented with the mutation-containing isoforms. Analysis of fractionated protein extracts by probing with affinity-purified antibodies revealed that both SpoIID and SpoIIP were present in a pool of membrane associated proteins, extracted from sporulating cells (Figure S10A, B). Signal arising from SpoIIP was also detected at similar levels in a soluble fraction of cell extract (Figure S10B, D); SpoIID however, presented differential detection pattern (Figure S10A, C). Noticeably lower amounts of SpoIID was observed for Zn-binding mutants compared to WT in the

membrane fraction of lysates, highlighting a potential role of the metal in protein stability *in vivo*, reflecting the *in vitro* observations (Figure S10C). A loss of signal in the soluble fraction of corresponding lysates (Figure S10C) further supports the hypothesis that lower stability of these isoforms leads to increased protein degradation and resulting reduced availability. Conversely, increased amount of protein was detected for the catalytic mutant of SpoIID (Δ*spoIID* complemented with *spoIID*$_{E101A}$) when compared to WT. This indicates accumulation of a more stable (Figure S10C) but inactive (Figure 7) form of the protein on the membrane of cells that are unable to complete engulfment and progress in sporulation (Figure 8).

Together, these results indicate that zinc and the residues involved in metal coordination are likely to play a structural, stabilizing role in SpoIID, although not all residues seem to be essential.

Protein expression in BACTH strains

To confirm protein expression and stability in the BACTH strains, immunoblotting with the relevant rabbit-raised antibodies was carried out for selected fusion combinations. A monoclonal anti-CyaA (adenylate cyclase, see main text for details) antibody recognising T18 fragment was used to cross-validate this immunoblot analysis. Combinations where only one of the fusion orientations (T18:25 or T25:T18) resulted in a detectable interaction were tested, as well as those described in the engulfment mechanism proposed in Figure 1A. Despite cross-reaction of the polyclonal antibodies with native *E. coli* proteins, particularly for anti-SpoIID and anti-SpoIIQ, as well as observed degradation issues, the combined information for the protein-specific and anti-CyaA antibodies allowed us to confirm the presence of all proteins in all investigated orientations, but at varying levels of expression and stability. The fact that some interactions are only detected in one combination suggests that protein orientation might also play an important role in establishing the correct engulfment machinery.

Immunoblotting revealed clear differences in protein stability and expression levels under analyzed conditions. Notably, fusion forms of SpoIIP$_{H142R}$ and SpoIIIAH seem to degrade, with the full

length $SpoIIP_{H142R}$ or SpoIIIAH protein being detected when probed with the respective antibody (Figure S7C, purple arrowheads; G, orange arrowheads, respectively). Importantly, probing with the anti-CyaA antibody confirms that the complete fusion is still present (D, purple arrowheads; H, orange arrowheads, respectively). This partial degradation could account for the weaker interactions detected for $SpoIIP_{H142R}$ and SpoIIIAH (Figure 5). SpoIIM is an integral membrane protein and no antibodies could be obtained but probing with anti-CyaA reveals the presence of the T18-SpoIIM fusions (light pink arrowheads, B and D) albeit at low levels, which could lead to the relatively low interactions observed in β-galactosidase assay. T18-SpoIID fusions were clearly visible when using anti-CyaA antibodies, despite some degradation (B, pink arrowheads), confirming our tentative identification of SpoIID when probing with anti-SpoIID antibodies (A, pink arrowheads). Finally, presence of SpoIIQ in the BACTH strains was confirmed combining information from immunoblots using anti-SpoIIQ (C, blue arrowheads) and anti-CyaA (D, blue arrowheads).

Supplementary experimental procedures

Circular dichroism

Circular dichroism (CD) data were collected for soluble SpoIID and SpoIIP recombinant proteins using a JASCO J-810 spectrophotometer and a 1 mm path length quartz cuvette (Hellma), where the temperature was maintained at 20°C, by a PTC-4235 Peltier temperature controller. Proteins were buffer exchanged into 50 mM Na_2HPO_4 pH 8.0, 50 mM NaF, using Satorious Vivaspin 500 centrifugal concentrators (10,000 MWCO), as per manufacturer's instructions. Measurements were acquired for proteins at a final concentration of approximately 0.01-0.02mg/ml as confirmed by Bradford assay under the following conditions: 2 nm bandwidth, 4 second response, over 260-185nm wavelengths at 0.5 nm pitch with a scanning speed of 50nm/min. Presented sSpoIID data are the result of 4 accumulations, whereas sSpoIIP scans are the average of 10 accumulations. Spectrum scan data were corrected to a buffer-only reference scan and 10-neighbour Savitzky-Golay smoothing (Savitzky

and Golay, 1964) was applied to final curves. Estimation of secondary structure composition was performed using the CDSSTR program, reference data set 4 (Sreerama and Woody, 2000) available via DichroWeb (Whitmore and Wallace, 2004).

Thermostability experiments were performed by monitoring a change of CD signal of proteins (approximately 0.2 mg/ml concentration in 50 mM Na_2HPO_4 pH 8.0, 50 mM NaF buffer) at 222 nm between 4 and 95 ^oC, at 1 ^oC /s, with 2 nm bandwidth, 4 second response time using the aforementioned cuvette. Mean residue ellipticity was calculated and the apparent melting temperature, T_m (°C), was determined from unfolding curves using the midpoint of the sigmoidal fit of the calculated MRE values using GraphPad Prism 7 software (La Jolla California USA, www.graphpad.com). The unfolding curves were normalized to give 0 for fully folded signal at 4 °C and 1 for fully unfolded signal at 90 °C.

Affinity purification of polyclonal antibodies from rabbit serum

Purified $SpoIID_{26\text{-}354}$ and $SpoIIP_{27\text{-}339}$ were used to immunize rabbits for polyclonal antibody production (Moravian Biotechnology). Carbonyldiimidazole (CDI)-activated crosslinked 6% beaded agarose resin (Pierce) was used for purification of antibodies from final bleed rabbit sera via affinity to column-immobilized immunogen. A sample of 0.5 ml of beads was washed with 100 mM borate pH 8.5 buffer to remove acetone storage solution and equilibrate the resin. An aliquot of immunogen protein at 1-5 mg/ml concentration in 100 mM borate pH 8.5 buffer was added to agarose and incubated for 24h at 4°C. Unbound protein was removed and resin was incubated at 4°C for 5h with 1 ml of 1M Tris pH 8.8 and washed with PBS 0.1% (v/v) Tween20. Agarose-immobilised protein was added to 15 ml of final bleed rabbit sera and incubated with mixing at 4°C for 18h. Serum was recovered, and agarose was washed with PBS 0.1% (v/v) Tween20. IgG elution was carried out with 0.1M glycine pH 2.5 in 5 consecutive steps of 30min incubation at room temperature, centrifugation 1 min at 3,000 x *g*, and neutralization of recovered supernatant with 0.1ml of 1M Tris pH 8.8. Concentration of purified antibodies was tested using NanoDrop 2000.

Detection of proteins using immunoblotting

Cell extracts or purified recombinant protein were normalized for protein concentration, resolved on 10 % and 12% SDS-PAGE gels, respectively, and transferred onto nitrocellulose membrane using Trans Blot Turbo (Bio-Rad) semi-dry transfer in Bjerrum Schafer-Nielsen buffer (48 mM Tris, 39 mM glycine, 20% methanol, pH 9.2). Following transfer, membrane was blocked for 1h (cell extracts) or overnight (recombinant protein) in 5% (w/v) milk TBS, 0.1% (v/v) Tween20, and incubated with affinity-purified rabbit polyclonal antibodies at 1:5000 dilution (cell extracts) or 1:15000 (recombinant protein) in 5% (w/v) milk TBS, 0.1% (v/v) Tween20 for 1-18h at 4 °C. Unbound primary antibodies were washed away from the membrane with three changes of TBS, 0.1% (v/v) Tween20. The membrane was probed with secondary anti-rabbit HRP-conjugated antibodies (Promega) at 1:2500 dilution in 5% (w/v) milk TBS, 0.1% (v/v) Tween20 for 1h at 4 °C. Membrane was washed three times with TBS, 0.1% (v/v) Tween20 and detection was carried out using Clarity ™ Western ECL Blotting Substrate (Bio-Rad) according to the manufacturer's protocol and visualized using Bio-Rad ChemiDoc XRS+ system.

Recombinantly expressed and purified proteins were prepared as detailed in the main text.

Whole cell extracts of BACTH strains were prepared from 2 ml samples of cultures grown in 10 ml LB broth, supplemented with carbenicillin (100 µg/ml), kanamycin (50 µg/ml) and 0.5 mM IPTG to an OD_{600} of 0.5. Cells were harvested by centrifugation (5 minutes at 17,000 x *g*), resuspended in 100 µl 20 mM Tris pH7.5 and 150 mM NaCl with the addition of 30 µl 4x Laemmli sample buffer and boiled for 10 minutes.

For detection of SpoIID and SpoIIP proteins in *C. difficile* cell extracts were prepared from sporulating cultures in 5ml SM broth, supplemented where necessary with 250 ng/ml of ATc (Ptet-spoIIP variants), and grown statically for 10h at 37 °C in a DG250 workstation (Don Whitley Scientific) under anaerobic conditions (10 % H_2, 10 % CO_2, 80 % N_2) as previously described (Fagan and Fairweather, 2011). All mutant and complemented strains were created in the 630Δ*erm*Δ*pyrE* background *via* allele-coupled exchange as described in the main text. Cells were harvested by

centrifugation (10 min at 4,000 x *g*), pellets were washed with 1 ml of PBS and frozen. Thawed cell pellets were resuspended in PBS containing protease inhibitors, 1.4 mg/ml lysozyme and 0.12 µg/ml DNaseI to an OD600 nm of 20 and incubated at 37°C for 1 h. Membranes were harvested by centrifugation at 21,100 *g* for 10 min at 4°C. The supernatant containing the soluble proteins was removed and mixed with an equal volume of 2 x Laemmli sample buffer. The harvested membranes were washed twice with 500 µl PBS, resuspended in PBS and solubilized with 1% SDS to a final equivalent OD600 nm of 20 and mixed with an equal volume of 2 x Laemmli sample buffer.

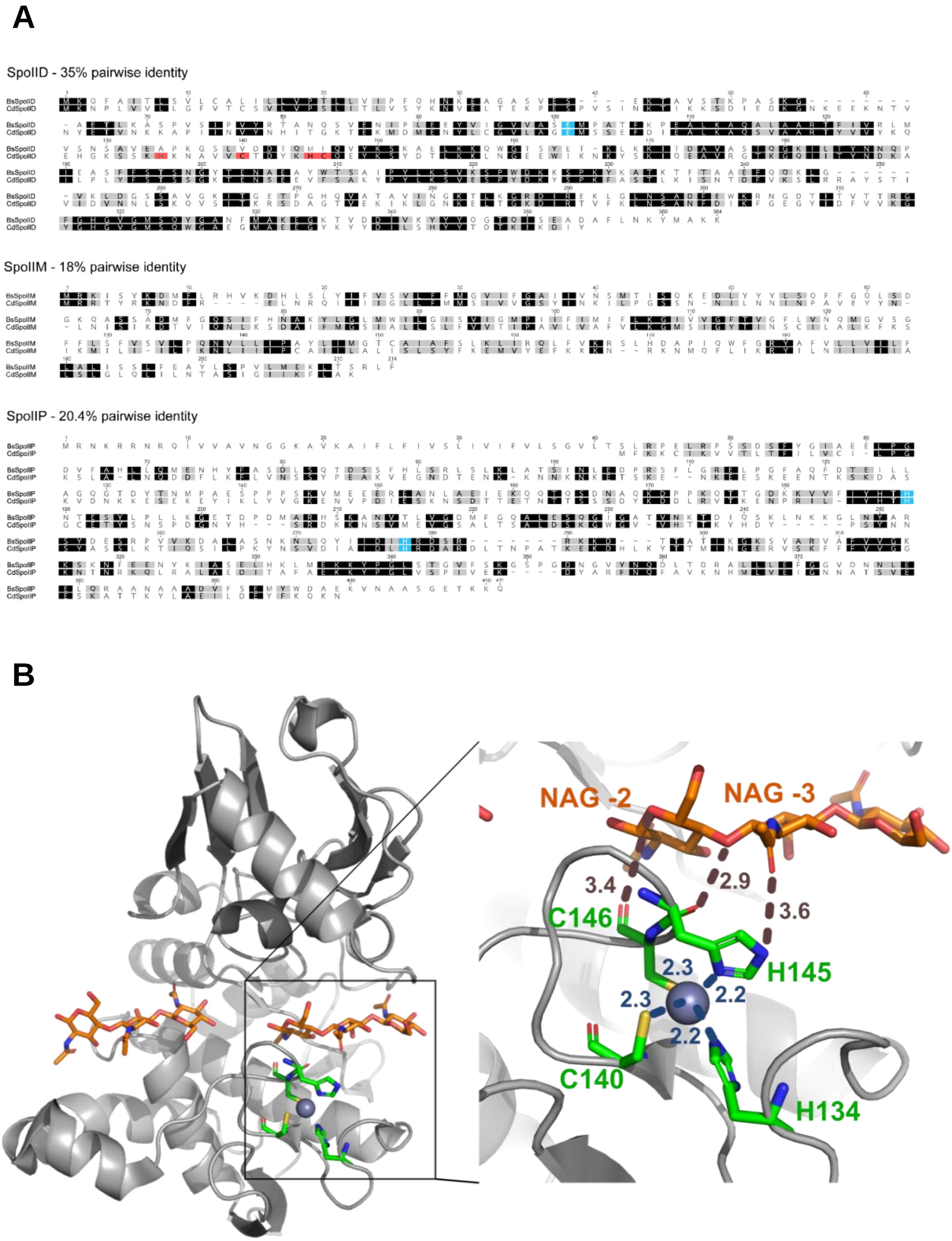
A
SpoIID - 35% pairwise identity
BsSpoIID
CdSpoIID
SpoIIM - 18% pairwise identity
BsSpoIIM
CdSpoIIM
SpoIIP - 20.4% pairwise identity
BsSpoIIP
CdSpoIIP
B
NAG -2
NAG -3
3.4
2.9
3.6
C146
H145
2.3
2.3
2.2
2.2
C140
H134

Figure S1. Conservation of SpoIIDMP sequences between *B. subtilis* and *C. difficile*

(A) Pairwise alignments of SpoIID, SpoIIM and SpoIIP sequences. Sequence identity is provided above each alignment. Conserved residues deemed essential for enzymatic activity are highlighted in blue. SpoIID residues that contribute to zinc coordination are highlighted in red. Alignment created using ClustalW (Thompson *et al.*, 1994) with a BLOSUM62 substitution matrix. (B) Cartoon representation of *C. difficile* SpoIID structure with bound $[NAG]_3$ ligand mimic (PDB accession code 5i1t, Nocadello *et al.*, 2016), highlighting the interactions between the zinc binding residues and the ligand (insert). H145 and C146 establish hydrogen bonds with the NAG acetyl groups in positions -2 and -3, respectively, via their main-chain carbonyl groups. H145 also establishes a weaker hydrogen bond with one of the NH atoms in the ring with an acetyl group in NAG -3. The zinc coordination sphere composed of H134, C140, H145 and C146 is also represented.

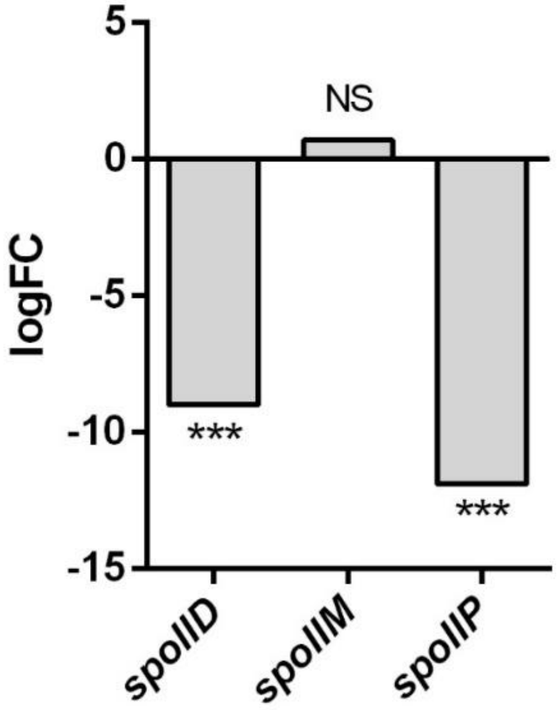

Figure S2. Forward genetic screen of *spoIIDMP* essentiality in *C. difficile* sporulation

A comprehensive transposon mutant library created in *C. difficile* R20291 was sporulated, analysed by Transposon Directed Insertion Site Sequencing (TraDIS) and compared to the input library (Dembek *et al.*, 2013). Genes with a log fold change (logFC) of transposon insertion sites > -2 were deemed required for sporulation. *** - p = 1.7e-45 (*spoIID*) and 1.9.3e-13 (*spoIIP*). This analysis indicates that *spoIID and spoIIP* are essential for effective sporulation, whilst *spoIIM* seems to be dispensable.

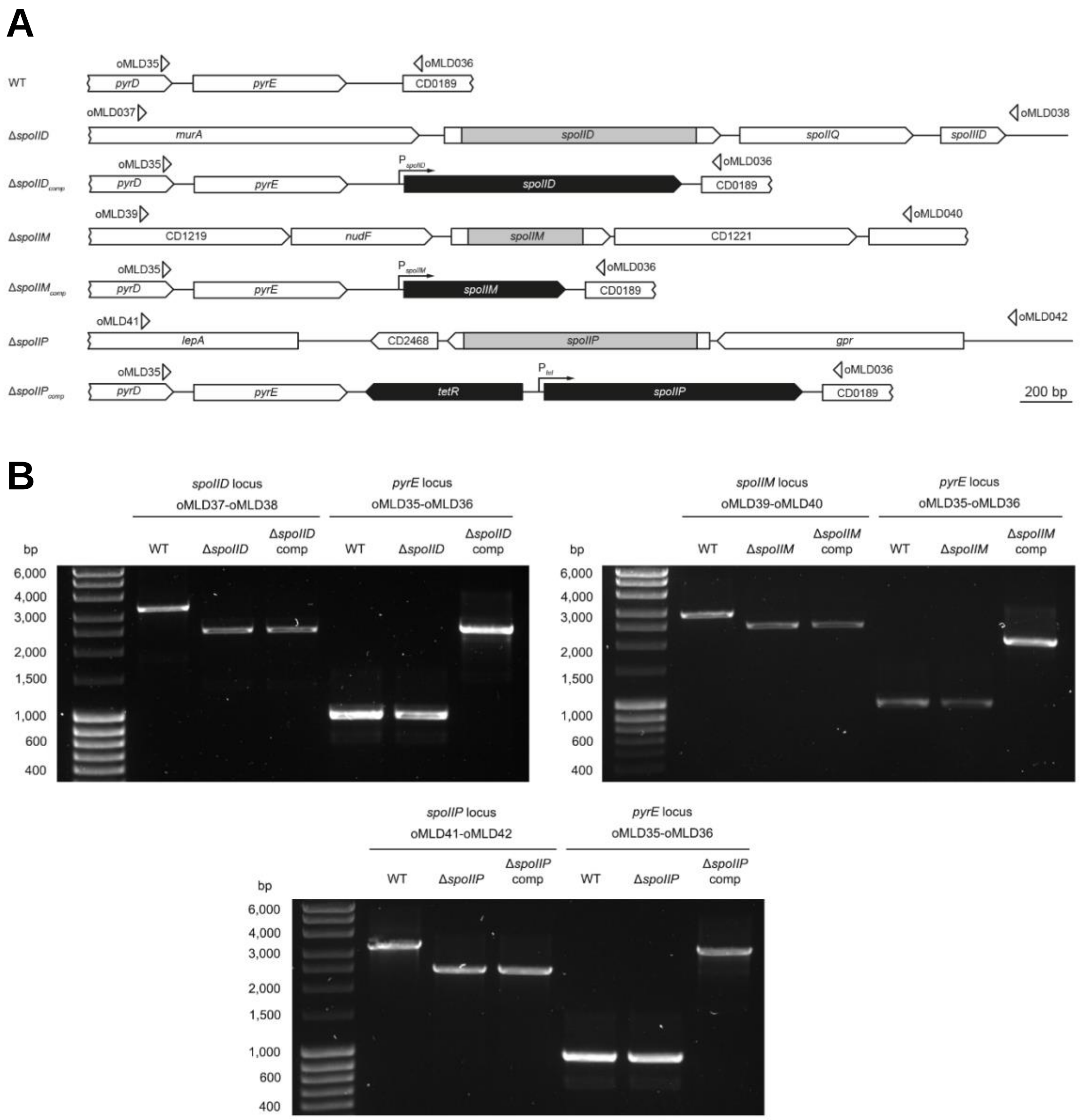

Figure S3. Construction of *spoIID, spoIIM* and *spoIIP* mutants in 630Δ*erm* by ACE

(A) Schematic representation of *spoIID*, *spoIIM, spoIIP* and *pyr*E loci in 630Δerm (WT), Δ*spoIID*, Δ$spoIID_{comp}$, Δ*spoIIM*, Δ$spoIIM_{comp}$, Δ*spoIIP* and Δ$spoIIP_{comp}$. Deleted regions are highlighted in grey. The resulting mutants were complemented by introduction of wild-type alleles of the genes (highlighted in black) downstream of *pyrE* under the control of native (*spoIID* and *spoIIM*) or inducible promoters (*spoIIP*). Binding sites for primers used in PCR screening are indicated with arrows (B) Agarose gel electrophoresis of PCR products obtained during mutant screening confirming correct integration of mutated alleles.

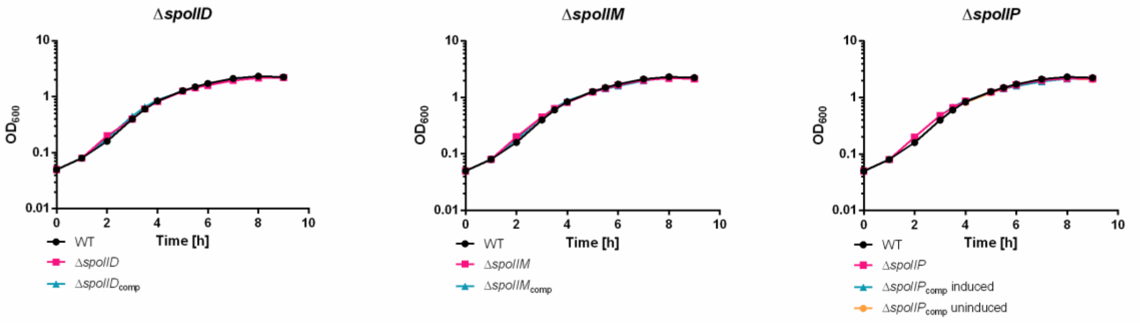

Figure S4. Growth curves of *spoIIDMP* mutants

Growth rate of Δ*spoIID*, Δ*spoIIM*, Δ*spoIIP* and corresponding complemented strains was compared to 630Δ*erm* (WT). All mutant strains exhibited vegetative growth equivalent to the WT strain. Overnight cultures were sub-cultured in BHIS to OD_{600} 0.05 and growth was monitored every hour by taking OD_{600} measurements. Data representative of experiments performed in biological triplicate.

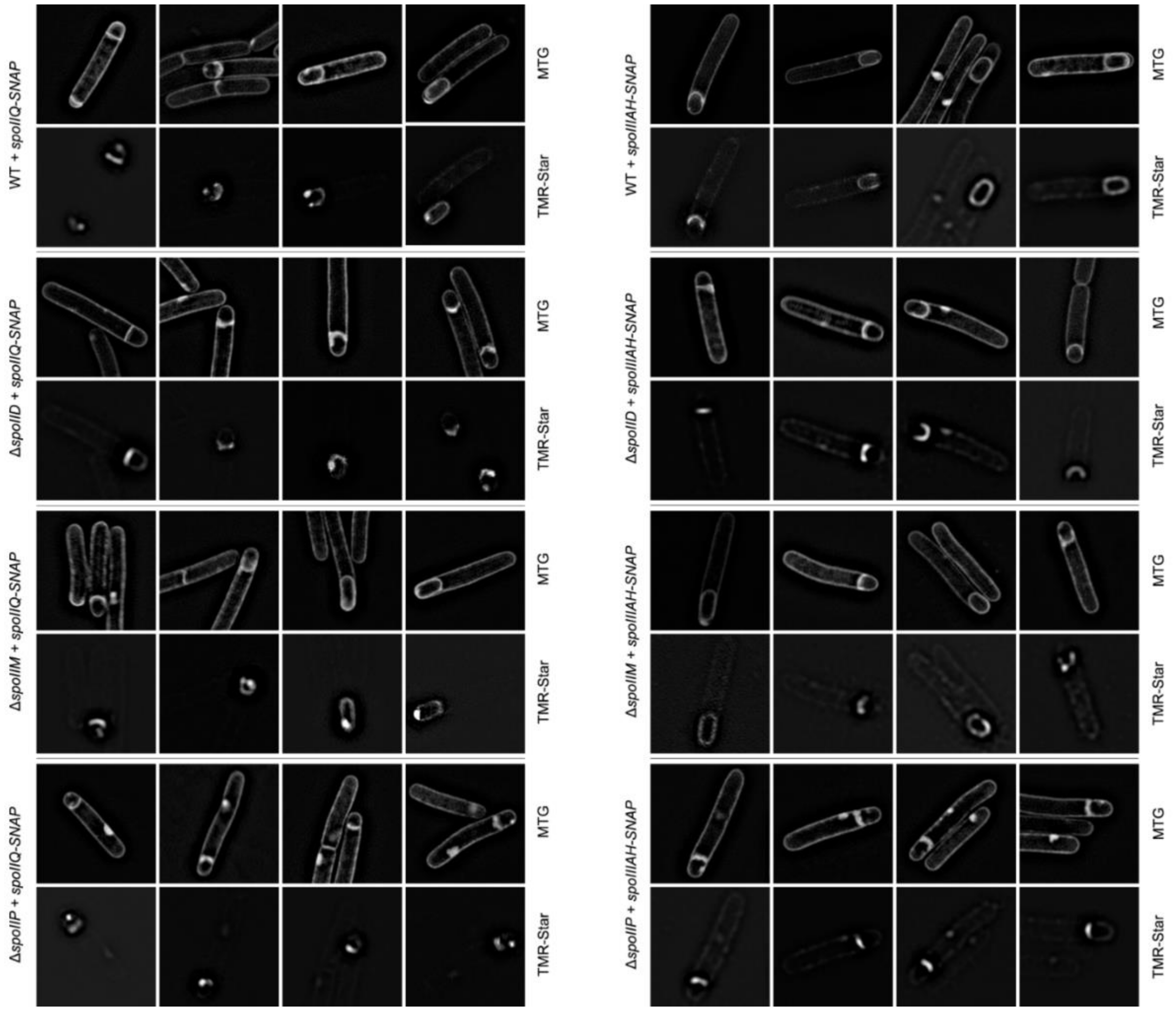

Figure S5. SIM analysis of SpoIIQ-SNAP and SpoIIIAH-SNAP localization in *spoIIDMP* mutants

In wild type and *spoIIM* mutants, SpoIIQ and SpoIIIAH follow the engulfing membrane as sporulation progresses. Both proteins localize at the flat septa or partially curved membrane seen in *spoIID* and *spoIIP* mutants as cells are arrested at early stages of engulfment. Although lack of D/P has no effect in the initial recruitment of SpoIIQ and SpoIIAH, a potential role in maintaining Q:AH localization later in the process cannot be excluded. SIM images of wild type and *spoIIDMP* mutant sporangia cells expressing either SpoIIQ (left) or SpoIIIAH (right) SNAP fusions, harvested after 14h of growth in SM broth and stained with MitoTracker Green (membrane) and TMR-Star (SNAP substrate). Scale bars corresponds to 2 µm. Images are representative of at least 3 biological replicates.

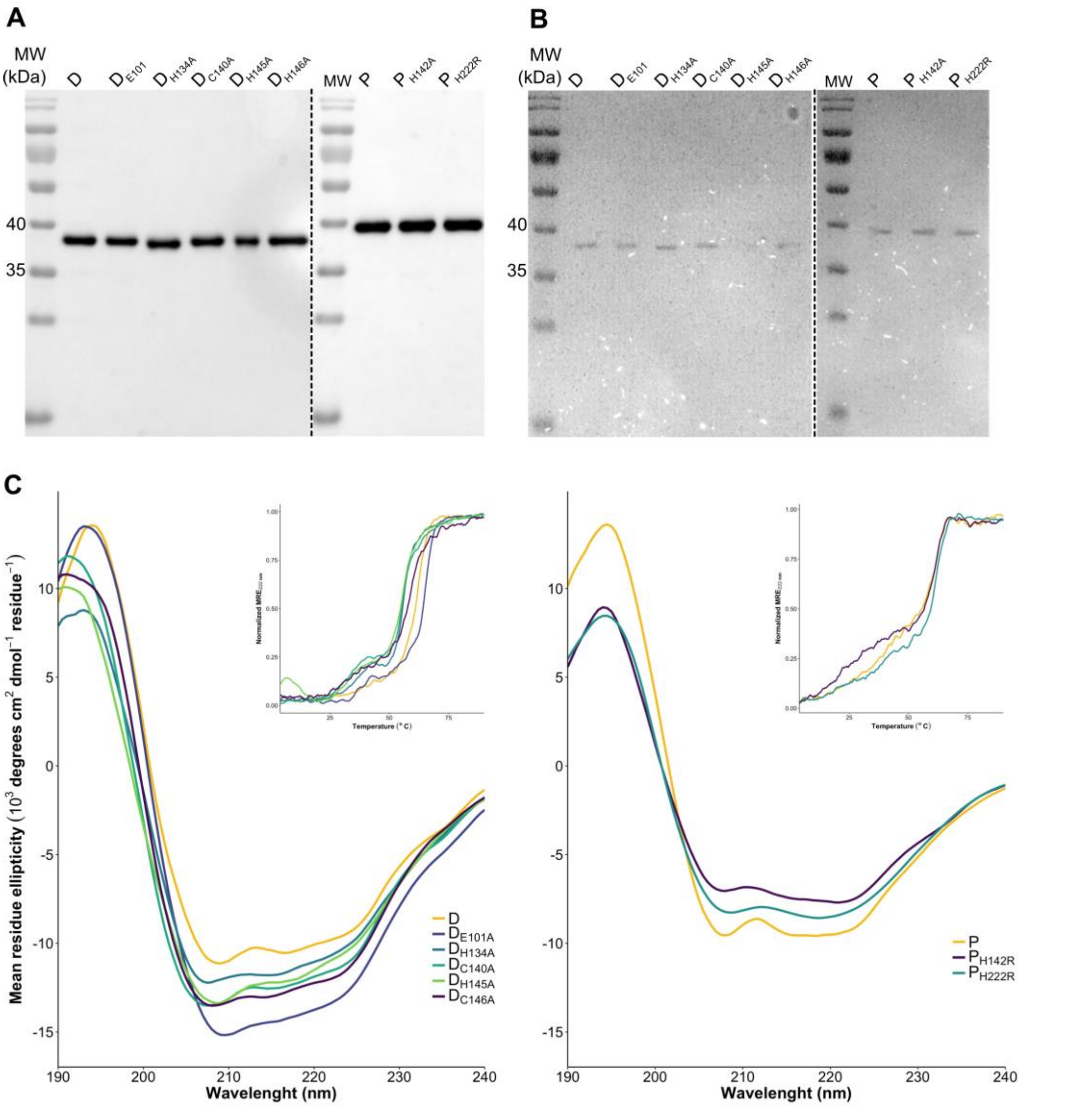

D

Protein	Method	Helix (%)	Strand (%)	Coil (%)	Unordered (%)	T_m(°C)
**sSpoIID**	PDB	34	17	-	-	
	PSIPRED	24	21	-	55	60.5
	CDSSTR	33	17	20	29	
**sSpoIID$_{E101A}$**	CDSSTR	36	18	20	26	63.4
**sSpoIID$_{H134A}$**	CDSSTR	35	17	22	29	55.3
**sSpoIID$_{C140A}$**	CDSSTR	33	17	21	29	54.5
**sSpoIID$_{H145A}$**	CDSSTR	28	19	22	31	54.5
**sSpoIID$_{C146A}$**	CDSSTR	33	17	21	29	57.1
**sSpoIIP**	PSIPRED	25	11	-	64	
	CDSSTR	30	20	20	29	53.7
**sSpoIIP$_{H142R}$**	CDSSTR	17	18	26	30	54.2
**sSpoIIP$_{H222R}$**	CDSSTR	16	18	25	31	57.8

Figure S6. Purity and stability of SpoIID$_{26-35}$ and SpoIIP$_{27-339}$ proteins

Proteins were purified to a high degree and no degradation was observed. Despite maintaining overall features and being folded in solution, the SpoIID zinc-binding mutants are less thermally stable. (A) Immunoblot analysis of purified SpoIID$_{25-354}$ and SpoIIP$_{27-339}$ isoforms and corresponding SDS-PAGE gel (B). Aliquots of 100 ng of purified proteins were resolved on two 12 % SDS-PAGE gels in parallel, with one subjected to immunoblot analysis using rabbit anti-SpoIID (left) and anti-SpoIIP (right) antibodies at a 1:15000 dilution. (C) Circular dichroism spectra of all variants of sSpoIID (left) and sSpoIIP (right). Inset corresponds to the melting curves, measured between 4 and 95 °C. (D) Secondary structure and thermal stability calculations. Assignment from CD data was carried out using the CDSSTR algorithm (Sreerama and Woody, 2000) at the DICHROWEB (Whitmore and Wallace, 2004) server and reference set 4. Secondary structure predictions from PSIPRED (Jones, 1999) and, for SpoIID, assignments based on the protein structure (PDB accession code 5i1t, Nocadello *et al.*, 2016) are presented. CDSSTR analysis for sSpoIID correlates well with the determined structure, but not with PSIPRED. Similarly, although sSpoIIP mutants have similar helical and strand composition based on CDSSTR analysis, this differs considerably from PSIPRED predictions.

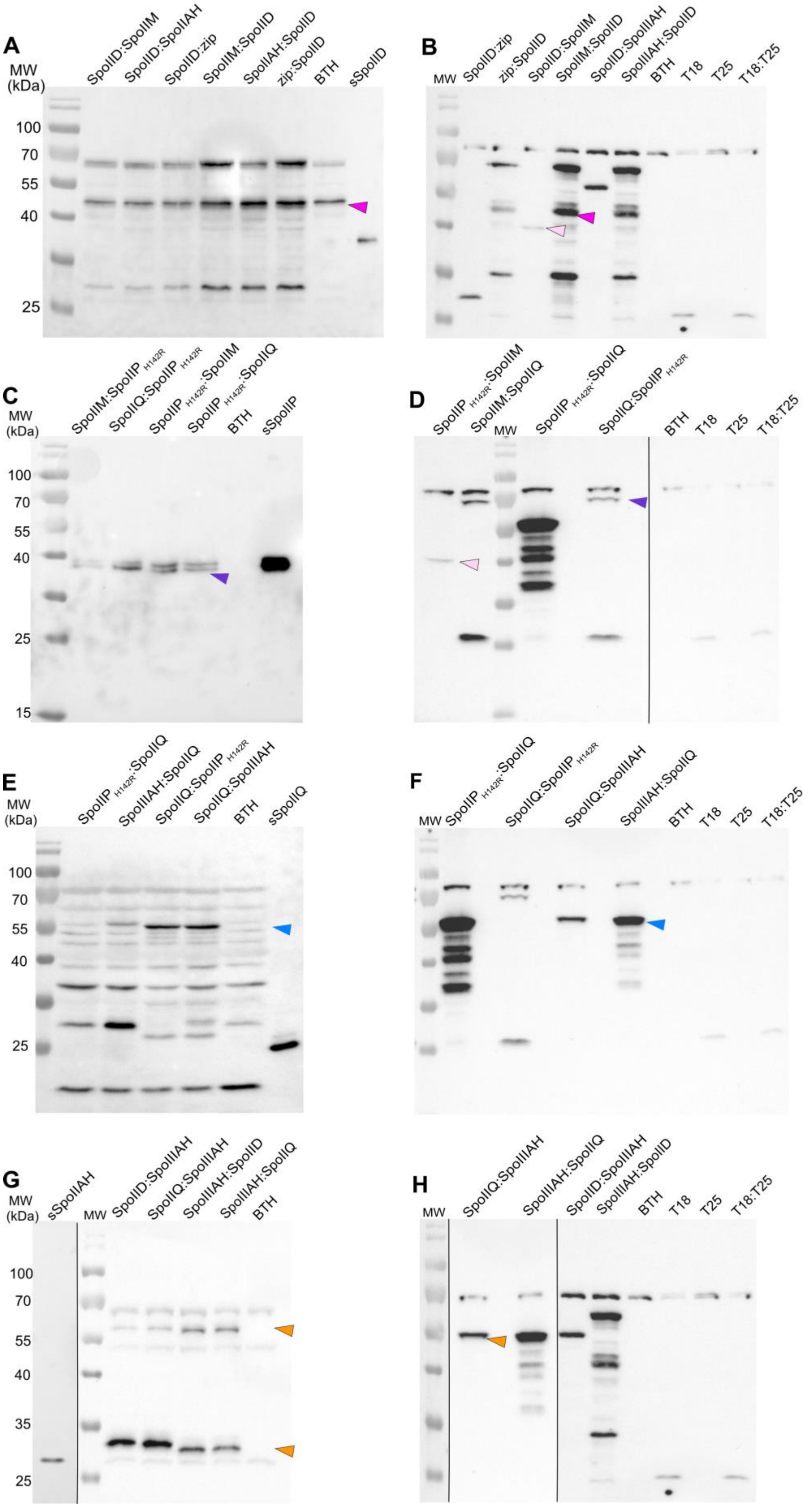

A
MW (kDa)
SpoIID:SpoIIM
SpoIID:SpoIIIAH
SpoIID:zip
SpoIIM:SpoIID
SpoIIIAH:SpoIID
zip:SpoIID
BTH
sSpoIID
100
70
55
40
25
B
MW
SpoIID:zip
zip:SpoIID
SpoIID:SpoIIM
SpoIIM:SpoIID
SpoIID:SpoIIIAH
SpoIIIAH:SpoIID
BTH
T18
T25
T18:T25
C
MW (kDa)
SpoIIM:SpoIIP H142R
SpoIIQ:SpoIIP H142R
SpoIIP H142R:SpoIIM
SpoIIP H142R:SpoIIQ
BTH
sSpoIIP
100
70
55
40
25
15
D
SpoIIP H142R:SpoIIM
SpoIIM:SpoIIQ
MW
SpoIIP H142R:SpoIIQ
SpoIIQ:SpoIIP H142R
BTH
T18
T25
T18:T25
E
MW (kDa)
SpoIIP H142R:SpoIIQ
SpoIIIAH:SpoIIQ
SpoIIQ:SpoIIP H142R
SpoIIQ:SpoIIIAH
BTH
sSpoIIQ
100
70
55
40
25
F
MW
SpoIIP H142R:SpoIIQ
SpoIIQ:SpoIIP H142R
SpoIIQ:SpoIIIAH
SpoIIIAH:SpoIIQ
BTH
T18
T25
T18:T25
G
MW (kDa)
sSpoIIAH
MW
SpoIID:SpoIIIAH
SpoIIQ:SpoIIIAH
SpoIIIAH:SpoIID
SpoIIIAH:SpoIIQ
BTH
100
70
55
40
35
25
H
MW
SpoIIQ:SpoIIIAH
SpoIIIAH:SpoIIQ
SpoIID:SpoIIIAH
SpoIIIAH:SpoIID
BTH
T18
T25
T18:T25

Figure S7. Immunoblot analysis of *E. coli* cell extracts used in BACTH

Presence of the different fusion proteins in the combination strains used in the BACTH assays was investigated using a combination of anti-SpoIID (A), anti-SpoIIP (C), anti-SpoIIQ (E) or anti-SpoIIIAH (G). The same strain combinations were tested using a monoclonal antibody against adenylate cyclase CyaA, which recognises the T18 fragment (B, D, F, H). All proteins tested were present in these strains, despite clear differences in protein amount and stability.

BACTH strains were grown to an OD_{600} of 0.5, the cells harvested and extracts resolved on 12% SDS-PAGE gels which were then subject to immunoblot analysis using rabbit anti-SpoIID (A), anti-SpoIIP (B), anti-SpoIIQ (C) or anti-SpoIIIAH (D) at a 1:5000 dilution, and an anti-rabbit secondary antibody conjugated to HRP was used at dilution of 1:2500. Secondary immunoblot analyses for the T18 fragments were conducted using mouse anti-CyaA conjugated to HRP (Santa Cruz) at a 1:200 dilution. Arrowheads indicate the bands corresponding to the different proteins being investigated: SpoIID - pink; SpoIIM - light pink; $SpoIIP_{H142R}$ - purple; SpoIIQ - blue; SpoIIIAH - orange.

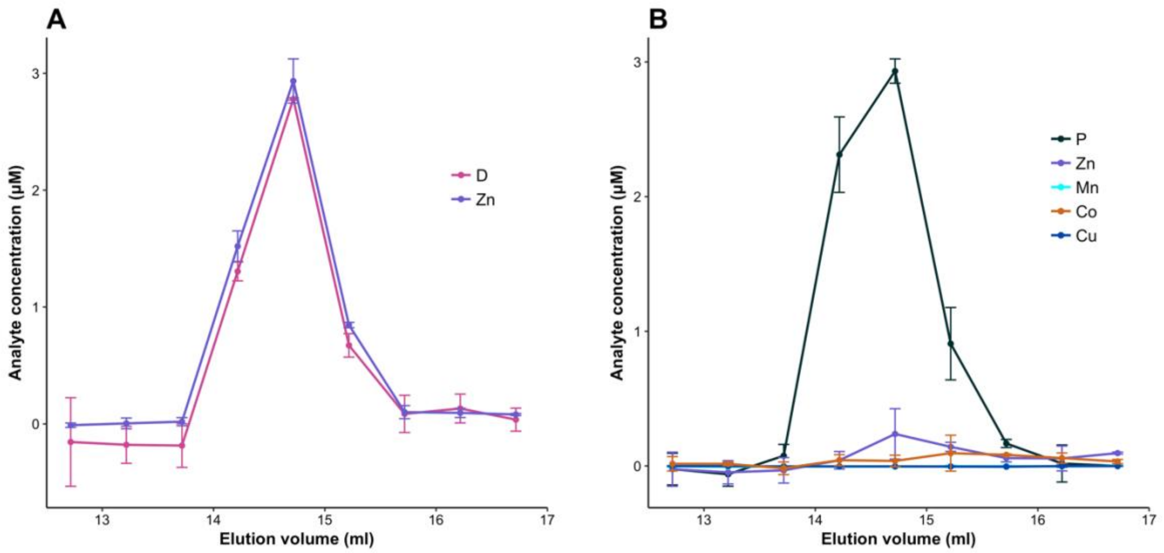

Figure S8. Metal content analysis of SpoIID$_{26\text{-}354}$ in the presence of EDTA and of SpoIIP$_{27\text{-}339}$

SpoIID retained zinc at an occupancy of 1:1 after incubation with 5mM EDTA, indicating that metal coordination is relatively strong, with 5 mM chelating agent not enough to remove it. Attempts to remove zinc by increasing EDTA concentration to 20mM resulted in protein precipitation, which indicates a possible structural role for the metal ion.

SpoIIP does not contain any of the metal divalent cations tested, frequently associated with enzymatic activity in metal binding proteins.

Purified protein was separated by SEC and resulting fractions analysed by ICP-MS for zinc (purple), manganese (cyan), copper (orange) and cobalt (blue) content. Absorbance at 280nm allowed protein concentration (dark green) determination, using theoretical MW of 37,680 Da and extinction coefficient of 48,380 $M^{-1} cm^{-1}$ for SpoIID$_{26\text{-}354}$ and MW of 35,950 Da and extinction coefficient of 30,830 $M^{-1} cm^{-1}$ for SpoIIP$_{27\text{-}339}$ (Expasy, ProtParam).

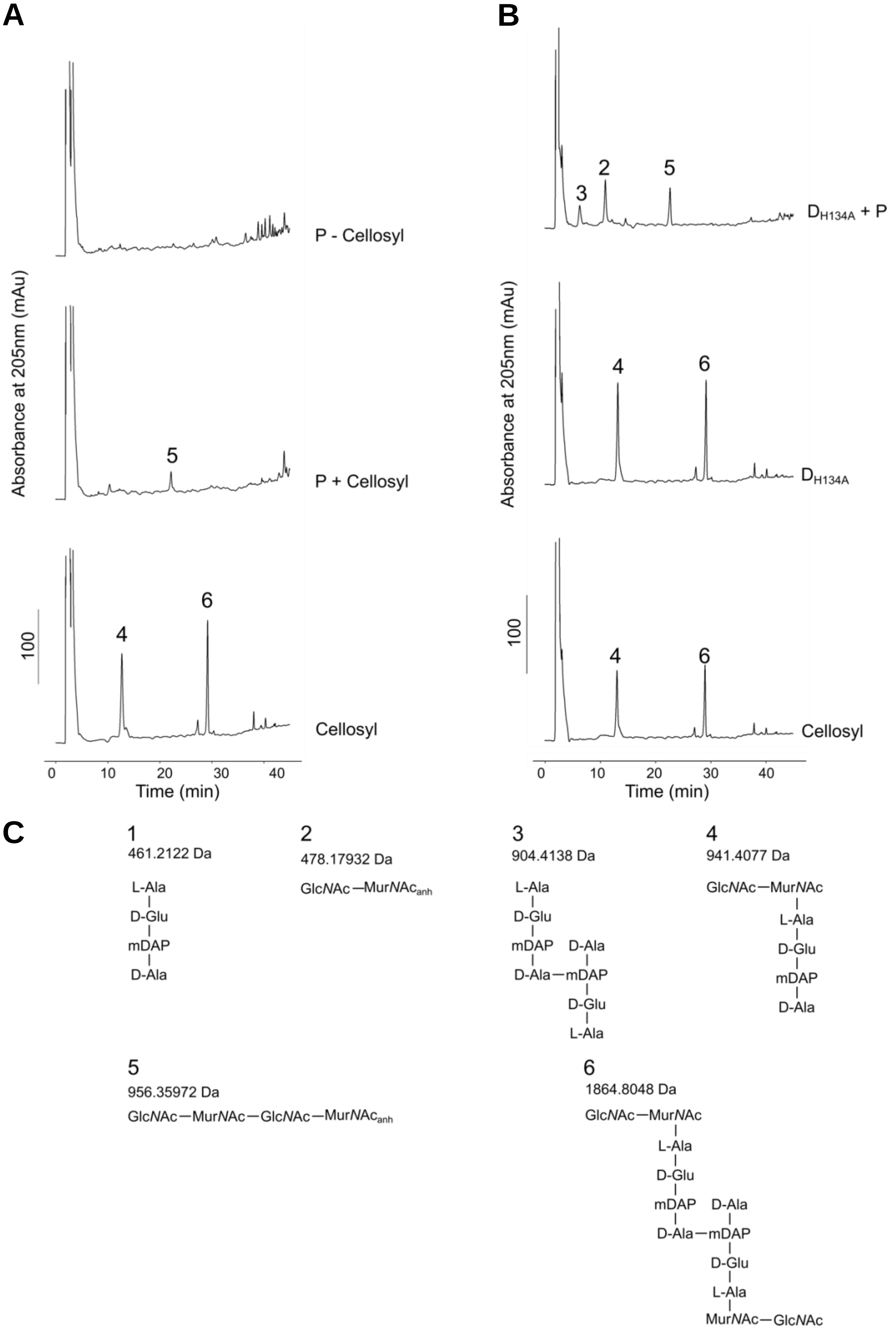
A
P - Cellosyl
5
P + Cellosyl
4
6
Cellosyl
100
Absorbance at 205nm (mAu)
0 10 20 30 40
Time (min)
B
3
2
5
D_H134A + P
4
6
D_H134A
100
Absorbance at 205nm (mAu)
Cellosyl
0 10 20 30 40
Time (min)
C
1
461.2122 Da
L-Ala
D-Glu
mDAP
D-Ala
2
478.17932 Da
GlcNAc—MurNAc_anh
3
904.4138 Da
L-Ala
D-Glu
mDAP D-Ala
D-Ala—mDAP
D-Glu
L-Ala
4
941.4077 Da
GlcNAc—MurNAc
L-Ala
D-Glu
mDAP
D-Ala
5
956.35972 Da
GlcNAc—MurNAc—GlcNAc—MurNAc_anh
6
1864.8048 Da
GlcNAc—MurNAc
L-Ala
D-Glu
mDAP D-Ala
D-Ala—mDAP
D-Glu
L-Ala
MurNAc—GlcNAc

Figure S9. Peptidoglycan degradation assays

Anhydro-disaccharide and anhydro-tetrasaccharide fragments detected when digesting PG with an active SpoIIP (Figure 7A, B, peaks 2 and 5), were no longer detectable when digesting PG with SpoIIP alone (A), indicating that they are a result of the amidase activity of SpoIIP acting on the naturally occurring anhydro-Mur*N*Ac glycan termini followed by cellosyl treatment.

H143A mutation does not completely abolish activity as a significant amount of anhydro-disaccharide (B, peak 2) is detected when treating PG with SpoIIDH143A and SpoIIP. This suggest that not all zinc-binding residues are essential for activity, and that H143 together with C146, seem to have a less significant role.

Enzymatic activity was assayed by analysing the products of PG digestion by the different proteins through LC-MS, as detailed in the main text, but using an LTQ mass spectrometer (Thermo). (A) *E. coli* BW25113Δ*lpp* PG was digested O/N with cellosyl, $SpoIIP_{27\text{-}339}$ followed by cellosyl and $SpoIIP_{27\text{-}339}$ without any subsequent treatment. The reaction products were separated and numbered peaks of interest identified by MS. Chromatograms represent, from bottom to top: cellosyl; SpoIIP + cellosyl; SpoII alone. (B) Analysis of $SpoIID_{H134A}$ lytic transglycosylase activity: control (cellosyl); $SpoIID_{H134A}$; $SpoIID_{H134A}$ + SpoIIP. (C) Proposed structures of muropeptides identified and numbered in the chromatograms in panels A and B. Theoretical neutral masses are given in brackets below the masses calculated from mass spectra.

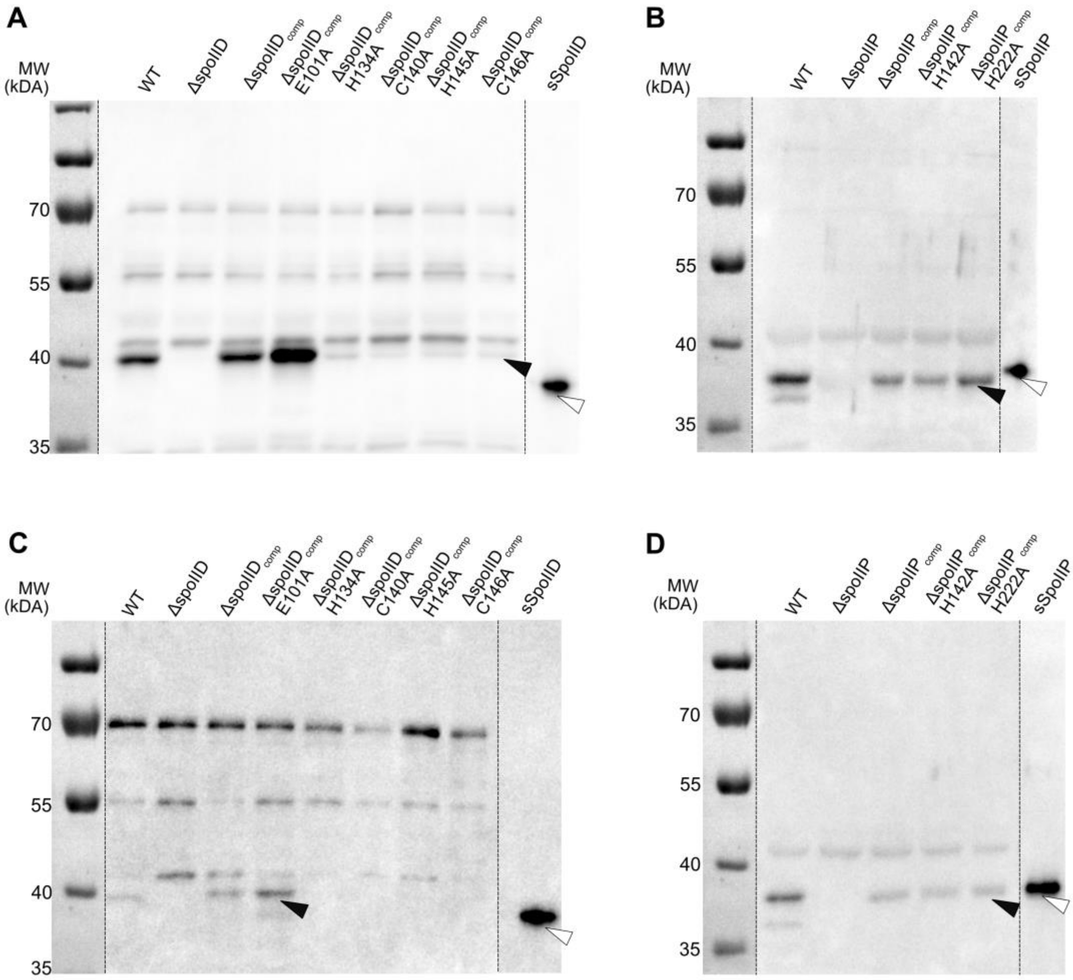

Figure S10. Immunoblot analysis of *C. difficile* SpoIID, SpoIIP and point mutant strains

Cell extracts were prepared from sporulating cells of *C. difficile* producing the variants of SpoIID (A, C) and SpoIIP (B, D) by fractionation into membrane and soluble proteins and resolved by 10% SDS-PAGE. Soluble recombinant SpoIID and SpoIIP proteins (indicated by white arrowhead) were used as an immunoblot positive control and molecular weight indicator for identification of proteins of interest (black arrowheads) in cell extracts. Polyclonal rabbit antibodies were used to detect the proteins of interest by probing fractionated extracts transferred onto nitrocellulose membrane at 1:5000 dilution in 5% milk, TBS, 0.1 % Tween20 solution. Secondary anti-rabbit antibodies conjugated to HRP (Promega; 1:2500) were detected using Clarity (TM) Western ECL Blotting Substrate (Bio-Rad) according to the manufacturer's protocol and visualized using Bio-Rad ChemiDoc XRS+ system. The position of molecular weight markers (kDa) is shown on the left side of each panel.

Table S1. Sporulation frequency

WT					
Time [h][a]	Total[b] [CFU/ml]		Spore[b] [CFU/ml]		Sporulation efficiency [%][c]
	Mean	SD	Mean	SD	
0	2.63E+08	7.64E+06	0.00E+00	0.00E+00	0.0000
6	2.90E+08	4.82E+07	0.00E+00	0.00E+00	0.0000
24	9.67E+07	2.25E+07	7.67E+03	1.04E+03	0.0029
48	2.88E+07	7.85E+06	1.40E+05	5.00E+03	0.0532
72	2.00E+07	2.29E+06	3.00E+05	1.32E+05	0.1139
96	1.72E+07	2.08E+06	4.17E+05	7.64E+04	0.1582
120	8.83E+06	1.76E+06	5.33E+05	2.89E+04	0.2025

Δ*spoIID*					
Time [h]	Total [CFU/ml]		Spore [CFU/ml]		Sporulation efficiency [%]
	Mean	SD	Mean	SD	
0	3.20E+08	2.65E+07	0.00E+00	0.00E+00	0.0000
6	3.48E+08	2.25E+07	0.00E+00	0.00E+00	0.0000
24	9.00E+07	2.50E+07	0.00E+00	0.00E+00	0.0000
48	2.88E+07	7.85E+06	0.00E+00	0.00E+00	0.0000
72	1.40E+07	1.80E+06	0.00E+00	0.00E+00	0.0000
96	1.03E+07	1.76E+06	0.00E+00	0.00E+00	0.0000
120	5.17E+06	2.36E+06	0.00E+00	0.00E+00	0.0000

Δ*spoIID*$_{comp}$					
Time [h]	Total [CFU/ml]		Spore [CFU/ml]		Sporulation efficiency [%]
	Mean	SD	Mean	SD	
0	2.88E+08	2.93E+07	0.00E+00	0.00E+00	0.0000
6	3.20E+08	5.41E+07	0.00E+00	0.00E+00	0.0000
24	8.00E+07	1.32E+07	4.00E+02	5.00E+01	0.0001
48	2.88E+07	7.85E+06	7.17E+04	7.64E+03	0.0249
72	9.17E+06	1.61E+06	1.02E+05	7.46E+04	0.0354
96	1.55E+07	4.27E+06	3.00E+05	5.00E+04	0.1040
120	7.83E+06	7.64E+05	2.83E+05	7.64E+04	0.0983

Δ*spoIIM*					
Time [h]	Total [CFU/ml]		Spore [CFU/ml]		Sporulation efficiency [%]
	Mean	SD	Mean	SD	
0	3.08E+08	1.26E+07	0.00E+00	0.00E+00	0.0000
6	3.25E+08	2.78E+07	0.00E+00	0.00E+00	0.0000
24	9.33E+07	2.89E+06	1.30E+03	1.80E+02	0.0004
48	2.88E+07	7.85E+06	1.25E+05	2.00E+04	0.0405
72	1.27E+07	2.31E+06	2.83E+05	2.89E+04	0.0919
96	1.30E+07	2.00E+06	2.17E+05	1.61E+05	0.0703
120	4.67E+06	7.64E+05	1.97E+05	6.81E+04	0.0638

Δ*spoIIM*$_{comp}$					
Time [h]	Total [CFU/ml]		Spore [CFU/ml]		Sporulation efficiency [%]
	Mean	SD	Mean	SD	
0	3.20E+08	2.65E+07	0.00E+00	0.00E+00	0.0000
6	3.48E+08	2.25E+07	0.00E+00	0.00E+00	0.0000
24	9.00E+07	2.50E+07	4.00E+02	5.00E+01	0.0001
48	2.88E+07	7.85E+06	7.17E+04	7.64E+03	0.0224
72	1.40E+07	1.80E+06	1.50E+05	1.00E+04	0.0469
96	1.03E+07	1.76E+06	3.00E+05	5.00E+04	0.0938
120	5.17E+06	2.36E+06	2.83E+05	7.64E+04	0.0885

Δ*spoIIP*					
Time [h]	Total [CFU/ml]		Spore [CFU/ml]		Sporulation efficiency [%]
	Mean	SD	Mean	SD	
0	3.55E+08	1.00E+07	0.00E+00	0.00E+00	0.0000
6	3.85E+08	4.92E+07	0.00E+00	0.00E+00	0.0000
24	5.33E+07	5.77E+06	0.00E+00	0.00E+00	0.0000
48	2.58E+07	2.02E+06	0.00E+00	0.00E+00	0.0000
72	1.60E+07	1.80E+06	0.00E+00	0.00E+00	0.0000
96	1.57E+07	3.18E+06	0.00E+00	0.00E+00	0.0000
120	7.33E+06	7.64E+05	0.00E+00	0.00E+00	0.0000

Δ*spoIIP*$_{comp}$ induced					
Time [h]	Total [CFU/ml]		Spore [CFU/ml]		Sporulation efficiency [%]
	Mean	SD	Mean	SD	
0	3.10E+08	8.66E+06	0.00E+00	0.00E+00	0.0000
6	3.67E+08	2.75E+07	0.00E+00	0.00E+00	0.0000
24	3.67E+07	7.64E+06	1.35E+04	3.61E+03	0.0044
48	2.57E+07	2.02E+06	4.67E+04	1.26E+04	0.0151
72	1.00E+07	5.00E+05	1.23E+05	1.26E+04	0.0398
96	8.83E+06	2.25E+06	1.63E+05	7.64E+03	0.0527
120	7.67E+06	1.26E+06	1.25E+05	2.50E+04	0.0403

a. Time in hours after reaching stationary phase
b. Values represent mean ± SD from experiment performed in biological triplicate
c. Sporulation efficiency expressed as percentage ratio of spore CFU at given time point divided by total CFU at t0

Table S2. Strains used in this study

Strain	Source	Relevant details
E. coli		
CA434	Purdy *et al*., 2002	Conjugation donor strain. HB101-derivative carrying the conjugative plasmid R702
BTH101	Euromedex	Reporter strain for BACTH
BL21 (DE3)	NEB	Protein overexpression strain for use with T7 promoter-based plasmids
C. difficile		
630	Hussain *et al,* 2005	Virulent, multidrug-resistant epidemic strain isolated in Zurich in 1982. Ribotype 012; *tcdA+*; *tcdB+*
630Δ*erm*	Hussain *et al,* 2005	Erythromycin-sensitive derivative of *C. difficile 630*
630Δ*erm*Δ*pyrE*	Ng *et al*, 2013	630Δerm carrying a mutated *pyrE* allele lacking 235 bp from the 3'-end of the gene.
630Δ*erm*Δ*pyrE*Δ*spoIID*	This study	630Δ*erm*Δ*pyrE* carrying a 900 bp deletion (aa 21-320) in *spoIID* (CD630_01240)
630Δ*erm*Δ*spoIID*	This study	630Δ*erm*Δ*pyrE*Δ*spoIID* with *pyrE* restored through homologous recombination with pMTL-YN1
630Δ*erm*Δ*spoIIM Pnat-spoIID*	This study	630Δ*erm*Δ*pyrE*Δ*spoIID* complemented with *spoIID* under the control of its native promoter and *pyrE* restored through homologous recombination with pMLD101
630Δ*erm*Δ*spoIIM Pnat-spoIID* E101A	This study	630Δ*erm*Δ*pyrE*Δ*spoIID* complemented with *spoIID* E101A under the control of its native promoter and *pyrE* restored through homologous recombination with pAXK003
630Δ*erm*Δ*spoIIM Pnat-spoIID* H134A	This study	630Δ*erm*Δ*pyrE*Δ*spoIID* complemented with *spoIID* H134A under the control of its native promoter and *pyrE* restored through homologous recombination with pAXK007

Strain	Source	Relevant details
630Δ*erm*Δ*spoIIM Pnat-spoIID* C140A	This study	630Δ*erm*Δ*pyrE*Δ*spoIID* complemented with *spoIID* C140A under the control of its native promoter and *pyrE* restored through homologous recombination with pAXK004
630Δ*erm*Δ*spoIIM Pnat-spoIID* H145A	This study	630Δ*erm*Δ*pyrE*Δ*spoIID* complemented with *spoIID* H145A under the control of its native promoter and *pyrE* restored through homologous recombination with pAXK005
630Δ*erm*Δ*spoIIM Pnat-spoIID* C146A	This study	630Δ*erm*Δ*pyrE*Δ*spoIID* complemented with spoIID C146A under the control of its native promoter and *pyrE* restored through homologous recombination with pAXK006
630Δ*erm*Δ*pyrE*Δ*spoIIM*	This study	630Δ*erm*Δ*pyrE* carrying a 450 bp deletion (aa 21-170) in *spoIID* (CD630_12210)
630Δ*erm*Δ*spoIIM*	This study	630Δ*erm*Δ*pyrE*Δ*spoIIM* with *pyrE* restored through homologous recombination with pMTL-YN1
630Δ*erm*Δ*spoIIM Pnat-spoIIM*	This study	630Δ*erm*Δ*pyrE*Δ*spoIIM* complemented with *spoIIM* under the control of its native promoter and *pyrE* restored through homologous recombination with pMLD102
630Δ*erm*Δ*pyrE*Δ*spoIIP*	This study	630Δ*erm*Δ*pyrE* carrying a 900 bp deletion (aa 21-320) in *spoIID* (CD630_24690)
630Δ*erm*Δ*spoIIP*	This study	630Δ*erm*Δ*pyrE*Δ*spoIIP* with *pyrE* restored through homologous recombination with pMTL-YN1
630Δ*erm*Δ*spoIIP Ptet-spoIIP*	This study	630Δ*erm*Δ*pyrE*Δ*spoIIP* complemented with *spoIIP* under the control of an inducible P*tet* promoter and *pyrE* restored through homologous recombination with pMLD142
630Δ*erm*Δ*spoIIP Ptet-spoIIP* H142R	This study	630Δ*erm*Δ*pyrE*Δ*spoIIP* complemented with *spoIIP* H142R under the control of an inducible P*tet* promoter and *pyrE* restored through homologous recombination with pMLD183
630Δ*erm*Δ*spoIIP Ptet-spoIIP* H222R	This study	630Δ*erm*Δ*pyrE*Δ*spoIIP* complemented with *spoIIP* H222R under the control of an inducible P*tet* promoter and *pyrE* restored through homologous recombination with pMLD184

Table S3. Primers used in this study

Name	Sequence (5’-3’)	Description
oMLD015	GGCGCGCCGCCATTATTTTTTTGAAC	pMTL-YN3 linearization
oMLD016	CCTGCAGGGGGCCCGATC	pMTL-YN3 linearization
oMLD017	CGGGCCCCCTGCAGGAGAAAATACACTTACTGTTGATGC	pMTL-YN3 -> *spoIID* LHR
oMLD018	CTCATACCTGATGGAACTAACACAGAGC	*spoIID* LHR <- *spoIID* RHR
oMLD019	GTTCCATCAGGTATGAGCCAGTGGGGA	*spoIID* LHR -> *spoIID* RHR
oMLD020	TAATGGCGGCGCGCCAACCTACATTTTTTTATGTTCCTTC	*spoIID* RHR <- pMTL-YN3
oMLD021	CGGGCCCCCTGCAGGAACTATAGGAAATGCGTTTG	pMTL-YN3 -> *spoIIM* LHR
oMLD022	ATATTTAGTATTATAATAATTATTTGTCTATTTAATTCTCTAAAATC	*spoIIM* LHR <- *spoIIM* RHR
oMLD023	ATAATTATTATAATACTAAATATTATAATTATAATAGCACTATCATTG	*spoIIM* LHR -> *spoIIM* RHR
oMLD024	TAATGGCGGCGCGCCTTAGGAATCTGAATATCTGGATATTC	*spoIIM* RHR <- pMTL-YN3
oMLD025	CGGGCCCCCTGCAGGCTTCATTATCTCCTTTAGGTGG	pMTL-YN3 -> *spoIIP* LHR
oMLD026	GCATTTTACCAAAGGCTACTACAAAGTATTTAGCC	*spoIIP* LHR <- *spoIIP* RHR
oMLD027	GTAGCCTTTGGTAAAATGCATACTAATATAAAAG	*spoIIP* LHR -> *spoIIP* RHR
oMLD028	TAATGGCGGCGCGCCAATAAATGATAGATAAGAAGGTTTACTG	*spoIIP* RHR <- pMTL-YN3
oMLD035	GAATAAAAAGTTTAGACGAAATAAGAGG	*pyrE* locus screening primer
oMLD036	TGCAAAAGTAGCTCCTACAGC	*pyrE* locus screening primer

Name	Sequence (5'-3')	Description
oMLD037	GACATGTAGGAGCAGAAGTTG	*pyrE spoIID* screening
oMLD038	TATTTCTTTTTATAATTTAATTCAATAAAAAAAGTAGG	*pyrE spoIID* screening
oMLD039	GATACTTTAAAAGAAAAAAAATTAATAGATAGTAC	*pyrE spoIIM* screening
oMLD040	ATCTATGTTTCCTATACCTAAATTTCTC	*pyrE spoIIM* screening
oMLD041	AAGAATCGAATATTAATGCCTTTAATGG	*pyrE spoIIP* screening
oMLD042	ATAAATAGTTGCTACCTTTTTCTGTTTG	*pyrE spoIIP* screening
oMLD065	GTAATAAGCGGCCGCTTATGTTGATATAGATAAGAAGATAACAG	P*nat-spoIID* complementation in pMTL-YN1C *via* Gibson Assembly
oMLD066	CGACTCTAGAGGATCCTTGACGTGTTTTTGGCATTTTATAC	P*nat-spoIID* complementation in pMTL-YN1C *via* Gibson Assembly
oMLD067	GTAATAAGCGGCCGCATAACATGGTATTAAAAAATGACGTTG	P*nat-spoIIM* complementation in pMTL-YN1C *via* Gibson Assembly
oMLD068	CGACTCTAGAGGATCCTATAAACTCCTTTATTTAGCTAGGAAC	P*nat-spoIIM* complementation in pMTL-YN1C *via* Gibson Assembly
oMLD133	GATCGCGGCCGCCATAAAAATAAGAAGCCTGCATTTGC	to clone P*tet* from pRPF185 NotI/SacI into pMTL-YN1C
oMLD134	GATCGAGCTCAGATCTGTTAACGCTAC	to clone P*tet* from pRPF185 NotI/SacI into pMTL-YN1C
oMLD212	GATCCCATGGGATCTTATAAAAATGTAGAATTAACTGAAAAACC	to clone *spoIID* (aa 26-354) into pET-M11 with an N-terminal TEV-cleavable His-Tag
oMLD213	GATCCTCGAGTTAGTATATATCTTTTATTTTTGTATCTGTGTAG	to clone *spoIID* (aa 26-354) into pET-M11 with an N-terminal TEV-cleavable His-Tag
oMLD214	GATCCTCGAGCTAATTTTTTTGTTTAAAATATTCATCCAAAATC	to clone *spoIIP* (aa 27-339) into pET-M11 with an N-terminal TEV-cleavable His-Tag

Name	Sequence (5'-3')	Description
oMLD215	GATCCCATGGGAAATCAAGATGATTTTTTAAAGTTTTTAGTAAATTC	to clone *spoIIP* (aa 27-339) into pET-M11 with an N-terminal TEV-cleavable His-Tag
oMLD244	GATCGGATCCCTAATTTTTTTGTTTAAAATATTCATCCAAAATC	to clone *spoIIP* CDS + RBS into pMTL-YN1T under the control of P*tet* via SacI/BamHI
oMLD245	GATCGAGCTCAGGAGGAGCAATTATGTTTAAAAAATG	to clone *spoIIP* CDS + RBS into pMTL-YN1T under the control of P*tet* via SacI/BamHI
oMLD295	GATCGGATCCGATGAAGAACCCATTGGTTGTTTTG	to clone full length (FL) *spoIID* into four BACTH vectors
oMLD296	GATCGGTACCCCGTATATATCTTTTATTTTTGTATCTGTGTAG	to clone FL *spoIID* into four BACTH vectors
oMLD297	GATCGGATCCGTTGAGAAGAACATATAGAAAAAATGATTTTAG	to clone FL *spoIIM* into four BACTH vectors
oMLD298	GATCGGTACCCCTTTAGCTAGGAACTTAATAATACCAATAC	to clone FL spoIIM into four BACTH vectors
oMLD299	GATCGGATCCGATGTTTAAAAAATGCATTAAAGTAGTAACC	to clone FL spoIIP into four BACTH vectors
oMLD300	GATCGGTACCCCATTTTTTTGTTTAAAATATTCATCCAAAATCTC	to clone FL spoIIP into four BACTH vectors
oMLD301	GATCGGATCCGATGAAGAAAAAGCTGTTAGAAAAAGATG	to clone FL spoIIQ into four BACTH vectors
oMLD302	GATCGGTACCGGCTTAATTAGACTCATTGGGTCTATAG	to clone FL spoIIQ into four BACTH vectors
oMLD303	GATCGGATCCGATGAAGTTTAATTATAAGGGAAGAGG	to clone FL spoIIIAH into four BACTH vectors
oMLD304	GATCGGTACCCCCTTATTACTATTATTATTTGTAAGTTTAATATTTTC	to clone FL spoIIIAH into four BACTH vectors
oMLD330	AGAGGATGTGAGACTTATTCAAATTC	to introduce H142R in SpoIIP *via* inverse PCR (equivalent to H189R in *B. subtilis*)

**Name**	**Sequence (5’-3’)**	**Description**
oMLD331	AGTATGATATATCAAAATTCTTGGATTTTC	to introduce H142R in SpoIIP *via* inverse PCR (equivalent to H189R in *B. subtilis*)
oMLD339	GCAATGTCTTCAGAGTTTGATATAGAAGC	to introduce E101A in SpoIID *via* inverse PCR
oMLD340	ACC AGC TAG TAC TCC ACA TAA ATA GTT C	to introduce E101A in SpoIID *via* inverse PCR
oMLD341	GCA ACT GAT TAT AAA CAT TGT CAA G	to introduce C140A in SpoIID *via* inverse PCR
oMLD342	TACTACTGCATTTTTATGTTTACTAG	to introduce C140A in SpoIID *via* inverse PCR
oMLD343	GCATGTCAAGAATATAAGAG	to introduce H145A in SpoIID *via* inverse PCR
oMLD344	TTTATAATCAGTACATACTACTG	to introduce H145A in SpoIID *via* inverse PCR
oMLD345	GCACAAGAATATAAGAGTTATG	to introduce C146A in SpoIID *via* inverse PCR
oMLD346	TGTTTATAATCAGTACATACTAC	to introduce C146A in SpoIID *via* inverse PCR
oMLD347	AGAAGAGATGCTAGGGATTTAAC	to introduce H222R in SpoIIP *via* inverse PCR (equivalent to H189R in *B. subtilis*)
oMLD348	TAAGTCTATCGCTATATCCACTG	to introduce H222R in SpoIIP *via* inverse PCR (equivalent to H189R in *B. subtilis*)
oAXK367	GCAAAAAATGCAGTAGTATGTACTG	to introduce H134A in SpoIID *via* inverse PCR
oAXK368	TTTACTAGATTTACCATGTTCTTGTTTATATAC	to introduce H134A in SpoIID *via* inverse PCR

Table S4. Plasmids used in this study

Plasmid	Descriptive name	Source	Relevant details
pMTL-YN3	pMTL8000-pCB102-*catP*-*pyrE*-ColE1+*tra*-T1-Z-T2	Ng *et al.*, 2013	Used to introduce deletions in 630Δ*erm*Δ*pyrE via* ACE
pMTL-YN1	pMTL8000-pCB102-*catP*-ColE1+*tra*-*pyrE*	Ng *et al.*, 2013	Used to restore *pyrE* to WT in 630Δ*erm*Δ*pyrE*
pMTL-YN1C	pMTL8000-pCB102-*catP*-ColE1+*tra*-*pyrE*-MCS	Ng *et al.*, 2013	Used to complement 630Δ*erm*Δ*pyrE*-derived mutants while restoring *pyrE* to WT
pMLD090	pMTL-YN3-Δ*spoIID*	This study	Used to introduce a 900 bp in-frame deletion (aa 21-320) in *spoIID* (CD630_01240)
pMLD091	pMTL-YN3-Δ*spoIIM*	This study	Used to introduce a 450 bp in-frame deletion (aa 21-170) in *spoIIM* (CD630_12210)
pMLD092	pMTL-YN3-Δ*spoIIP*	This study	Used to introduce a 900 bp in-frame deletion (aa 21-320) in *spoIIP* (CD630_24690)
pMLD101	pMTL-YN1C-P*nat*-*spoIID*	This study	Used to complement 630Δ*erm*Δ*pyrE*Δ*spoIID* with *spoIID* under the control of its native promoter while restoring *pyrE* to WT
pMLD102	pMTL-YN1C-P*nat*-*spoIIM*	This study	Used to complement 630Δ*erm*Δ*pyrE*Δ*spoIIM* with *spoIIM* under the control of its native promoter while restoring *pyrE* to WT
pMLD142	pMTL-YN1C-P*tet*-*spoIIP*	This study	Used to complement 630Δ*erm*Δ*pyrE*Δ*spoIIP* with *spoIIP* under the control of an inducible P*tet* promoter while restoring *pyrE* to WT
pMLD114	pFT58-spoIIQ	This study	SpoIIQ C-terminal SNAP fusion under the control of its native promoter
pMS481	pFT58-spoIIIAH	Serrano *et al.*, 2016	SpoIIIAH C-terminal SNAP fusion under the control of its native promoter

Plasmid	Descriptive name	Source	Relevant details
pMLD154	pUT18C-spoIID	This study	FL SpoIID fused to the C-terminus of adenylate cyclase T18 fragment *via* BamHI/KpnI cloning
pMLD155	pKT25-spoIID	This study	FL SpoIID fused to the C-terminus of adenylate cyclase T25 fragment *via* BamHI/KpnI cloning
pMLD156	pUT18C-spoIIM	This study	FL SpoIIM fused to the C-terminus of adenylate cyclase T18 fragment *via* BamHI/KpnI cloning
pMLD157	pKT25-spoIIM	This study	FL SpoIIM fused to the C-terminus of adenylate cyclase T25 fragment *via* BamHI/KpnI cloning
pMLD158	pUT18C-spoIIP H142R	This study	FL SpoIIP H142R fused to the C-terminus of adenylate cyclase T18 fragment *via* BamHI/KpnI cloning
pMLD159	pKT25-spoIIP	This study	FL SpoIIP H142R fused to the C-terminus of adenylate cyclase T25 fragment *via* BamHI/KpnI cloning
pMLD160	pUT18C-spoIIQ	This study	FL SpoIIQ fused to the C-terminus of adenylate cyclase T18 fragment *via* BamHI/KpnI cloning
pMLD161	pKT25-spoIIQ	This study	FL SpoIIQ fused to the C-terminus of adenylate cyclase T25 fragment *via* BamHI/KpnI cloning
pMLD162	pUT18C-spoIIIAH	This study	FL SpoIIIAH fused to the C-terminus of adenylate cyclase T18 fragment *via* BamHI/KpnI cloning
pMLD163	pKT25-spoIIIAH	This study	FL SpoIIIAH fused to the C-terminus of adenylate cyclase T25 fragment *via* BamHI/KpnI cloning
pMLD183	pMTL-YN1C-Ptet-*spoIIP* H142R	This study	H142R mutation in pMLD142 introduced *via* inverse PCR
pMLD184	pMTL-YN1C-Ptet-*spoIIP* H222R	This study	H222R mutation in pMLD142 introduced *via* inverse PCR

**Plasmid**	**Descriptive name**	**Source**	**Relevant details**
pAXK001	pETM-11-spoIID$_{26\text{-}354}$	This study	SpoIID$_{26\text{-}354}$ expression plasmid
pAXK002	pETM-11-spoIIP$_{27\text{-}339}$	This study	SpoIIP$_{27\text{-}339}$ expression plasmid
pAXK003	pMTL-YN1C-Pnat-*spoIID* E101A	This study	E101A mutation in pMLD101 introduced *via* inverse PCR
pAXK004	pMTL-YN1C-Pnat-*spoIID* C140A	This study	C140A mutation in pMLD101 introduced *via* inverse PCR
pAXK005	pMTL-YN1C-Pnat-*spoIID* H145A	This study	H145A mutation in pMLD101 introduced *via* inverse PCR
pAXK006	pMTL-YN1C-Pnat-*spoIID* C146A	This study	C146A mutation in pMLD101 introduced *via* inverse PCR
pAXK007	pMTL-YN1C-Pnat-*spoIID* H134A	This study	H134A mutation in pMLD101 introduced *via* inverse PCR
